# Systematic Review of NMR-Based Metabolomics Practices in Human Disease Research

**DOI:** 10.3390/metabo12100963

**Published:** 2022-10-12

**Authors:** Katherine Huang, Natalie Thomas, Paul R. Gooley, Christopher W. Armstrong

**Affiliations:** Department of Biochemistry and Pharmacology, Bio21 Molecular Science and Biotechnology Institute, University of Melbourne, Parkville, VIC 3010, Australia

**Keywords:** NMR spectroscopy, metabolomics, workflow, variation, standardisation

## Abstract

Nuclear magnetic resonance (NMR) spectroscopy is one of the principal analytical techniques for metabolomics. It has the advantages of minimal sample preparation and high reproducibility, making it an ideal technique for generating large amounts of metabolomics data for biobanks and large-scale studies. Metabolomics is a popular “omics” technology and has established itself as a comprehensive exploratory biomarker tool; however, it has yet to reach its collaborative potential in data collation due to the lack of standardisation of the metabolomics workflow seen across small-scale studies. This systematic review compiles the different NMR metabolomics methods used for serum, plasma, and urine studies, from sample collection to data analysis, that were most popularly employed over a two-year period in 2019 and 2020. It also outlines how these methods influence the raw data and the downstream interpretations, and the importance of reporting for reproducibility and result validation. This review can act as a valuable summary of NMR metabolomic workflows that are actively used in human biofluid research and will help guide the workflow choice for future research.

## 1. Introduction

Metabolomics aims to identify and measure metabolite snapshots of biospecimens that are representative of the biological condition of a subject, inclusive of internal and external factors. The dynamic metabolome is sensitive to perturbation of the subject’s state, and therefore requires the processes of metabolomics research to be well considered, precise, and rapid. Furthermore, for multiple snapshots within or between studies to be compared, the metabolomics process requires reproducible analytical instruments, standardised collection and acquisition procedures, robust statistical workflows, and minimal manual sample handling (automation).

The two main analytical instruments used for metabolomics are mass spectrometry (MS), coupled to gas or liquid chromatography, and nuclear magnetic resonance (NMR) spectroscopy. The advantages and disadvantages for each technique have been detailed previously [1]. MS is more widespread and commonly used, likely due to the improved sensitivity that enables more metabolites to be measured using both targeted and untargeted approaches. However, NMR offers key advantages in reproducibility for data acquisition, requires less sample preparation steps and allows for absolute quantitation that makes it favourable for data comparison across studies. It is also faster, and the methods are more economical than MS-based metabolomics experiments, making it ideal for large sample sizes. 

Metabolomics has become an attractive method for exploring human health, especially disease states, however, challenges remain in the direct comparison and replication of results between studies. Previous reviews have reported best practices for NMR metabolomics [2,3,4,5], and emphasized the importance of standardised reporting among metabolomic studies [6,7,8]. However, it is unknown how common these practices are employed in research settings and whether the reporting guidelines have been followed. Real-world evidence of the variation and popularity of workflows being used would provide useful guidance for researchers to decide on the right NMR-based workflow to apply to their disease of interest.

To this end, we systematically summarised NMR-based metabolomics workflows of all studies reported on human disease patients in 2019 and 2020. In this work we summarised 131 articles with the aim to reveal common NMR metabolomics practices employed in the literature, the variations at each workflow step and their impacts, comparability of data across studies, and adherence to reporting standards. 

## 2. Materials and Methods 

### 2.1. Protocol and Registration 

For this research we could not preregister the study in PROSPERO as it did not measure any health-related outcomes. The concept of this research was to identify variations and inconsistencies in the steps of NMR metabolomics workflow. We first critically analysed the first ten eligible articles in our literature search and extracted topics regarding different steps in the NMR metabolomics workflow that were to be examined. We then iteratively read and entered the corresponding information into columns for the rest of the articles. From our knowledge no such review exists that relates NMR-based metabolomics pitfalls supported by evidence from actual studies. 

### 2.2. Eligibility Criteria, Information Sources and Search Parameters

The search terms were “nuclear magnetic resonance”, “metabolomics” and “patients” in the title/abstract of papers published in 2019 and 2020 and included all studies that analysed human biofluids with NMR metabolomics. The search was conducted on the 8 August 2021 in a single database: PUBMED database. 

### 2.3. Study Selection 

We exported the search results into EndNote. Abstracts were assessed independently by KH and NT to remove articles that were non-human, reviews, method papers or involved in vivo NMR experiments. Full texts of publications were checked by KH to confirm eligibility criteria. After the identification of eligible articles, if articles investigated multiple biofluids, they were further separated into their own study. In this research an “article” refers to the entire publication and “studies” refers to the individual biofluid workflows. The final literature search was filtered for biofluids that were serum, plasma, or urine.

### 2.4. Data Collection Process and Data Items 

For each included study KH extracted the data in Excel. If an article referenced a protocol paper, the details reported in the protocol paper was compiled and referenced. For all included studies CA checked if the data extraction was performed correctly. Additionally, the following variables were also extracted but were not included into the results synthesis: (1) demographics of cohorts, (2) storage conditions for samples, (3) univariate analyses performed for clinical data, (4) pathways analysis, (5) software used for data analysis.

### 2.5. Synthesis of Results

No meta-analyses were predefined at the study conception stage, as the number of topics analysed, and the factors explored as determinants of articles addressing a topic could only be assessed after conducting the systematic review. The data extracted from this systematic review were all qualitative; bivariate (presence/absence) or non-ordinal categorical variables. Analysis began with generating frequency tables for each individual topic to identify initial trends. Topics were then analysed based on the flow of a typical NMR metabolomics workflow. Finally, topics were analysed holistically to visualise the different points of diversions that can occur across the NMR workflow. All data visualisations, frequency tables and percentages were constructed and calculated using the “R” statistical environment [9].

### 2.6. Risk of Bias

We are not aware of any tools that measure the risk of bias for this type of systematic review. All eligible studies gathered under the inclusion criteria were used in the result synthesis. Extracted information were validated by one or more researcher. 

## 3. Results 

### 3.1. Study Selection and Data Collected

**Figure 1:**
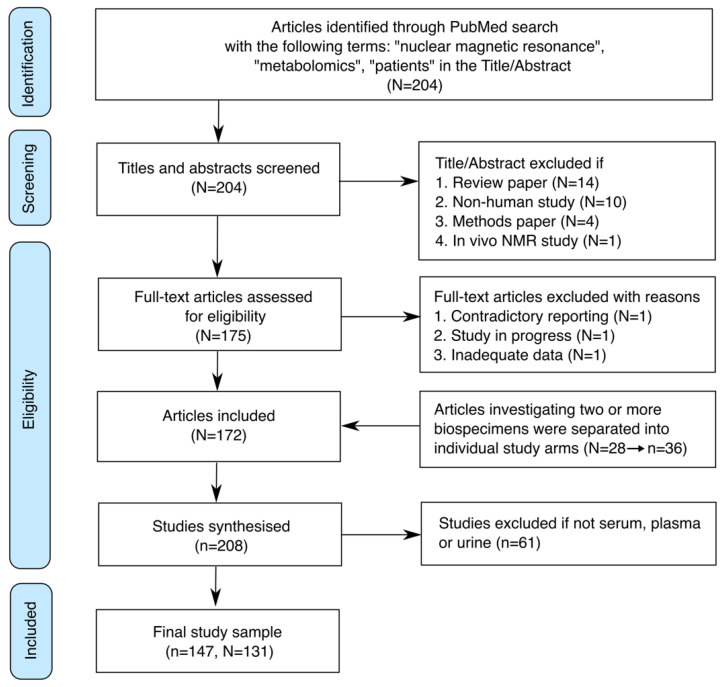
Preferred reporting items for systematic reviews and meta-analyses (PRISMA) flow diagram. After screening 204 articles, we synthesised 147 studies analysing NMR metabolomics workflows. N = number of articles, n = number of individual study arms.

We identified 172 articles published over a two-year period (2019 and 2020) that used NMR metabolomics to analyse biospecimens from human patients [10,11,12,13,14,15,16,17,18,19,20,21,22,23,24,25,26,27,28,29,30,31,32,33,34,35,36,37,38,39,40,41,42,43,44,45,46,47,48,49,50,51,52,53,54,55,56,57,58,59,60,61,62,63,64,65,66,67,68,69,70,71,72,73,74,75,76,77,78,79,80,81,82,83,84,85,86,87,88,89,90,91,92,93,94,95,96,97,98,99,100,101,102,103,104,105,106,107,108,109,110,111,112,113,114,115,116,117,118,119,120,121,122,123,124,125,126,127,128,129,130,131,132,133,134,135,136,137,138,139,140,141,142,143,144,145,146,147,148,149,150,151,152,153,154,155,156,157,158,159,160,161,162,163,164,165,166,167,168,169,170,171,172,173,174,175,176,177,178,179,180]. The screening process is shown in Figure 1. The 172 articles were divided into separate studies for each biospecimen type. Twenty-eight articles had investigated two or more biospecimens, producing 208 studies after breaking down biospecimens into their own individual workflow. These were then filtered for biofluids that were either serum, plasma, or urine. The final literature sample consisted of 131 articles with 147 studies. The studies of other biospecimens (n = 61) were excluded due to inadequate data for comparison. 

**Figure 2:**
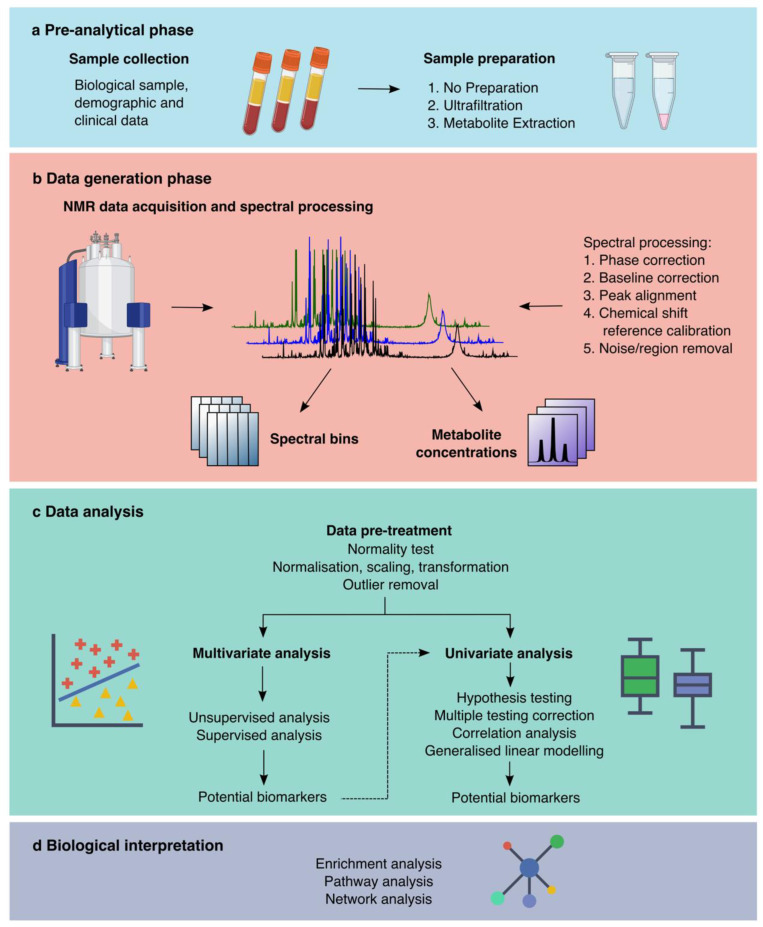
A typical workflow for untargeted NMR metabolomics. The NMR metabolomics workflow is divided into four main phases: (**a**) pre-analytical including sample collection and sample preparation, (**b**) data generation which involves NMR spectra acquisition, spectral processing, and the generation of spectral bins and/or metabolite concentrations. (**c**) Data analysis is performed for both data types including pre-treatment, multivariate and univariate analysis. The dashed arrow shows the optional integration of multivariate and univariate analyses. The fourth phase is (**d**) biological interpretation.

The core steps in an NMR metabolomics workflow include the pre-analytical phase, data generation, data analysis and biological interpretation (Figure 2). Briefly, the pre-analytical phase includes sample collection and sample preparation, which may include metabolite extraction or removal of macromolecules. Once the sample is prepared data generation is conducted by data acquisition, with the appropriate experimental parameters, spectral processing and then data can be presented as discrete bins that represent the area under the spectrum or individual metabolite concentrations. Data analysis includes data pre-treatment, analysis, and the identification of significant metabolites. Finally, biological interpretation provides biological context to the significant metabolites. 

**Figure 3:**
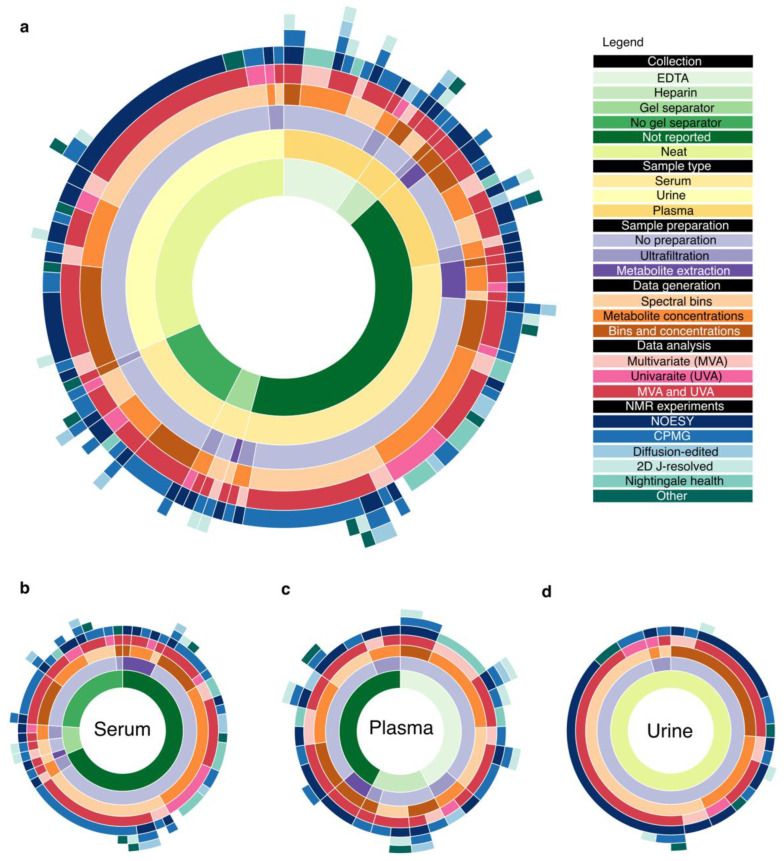
Sunburst charts showing the variation in workflows for NMR metabolomics. Each ring shows a step in the NMR metabolomics workflow and the segments show the percentage of studies that had used each method represented in (**a**) the whole literature search, (**b**) serum studies, (**c**) plasma studies and (**d**) urine studies only. The flavours of sunbursts can be explored interactively at https://rpubs.com/sunburstNMRMetabolomics accessed on 25 May 2022. One plasma study (Article ID: 99) that used sodium-citrate collection tube was removed in the visualization to keep the segments at a perceivable size (see Supplementary Data for the detailed workflow).

For the purposes of this review, we focused on all the main factors that were involved in the pre-analytical phase: (1) biofluid type, (2) collection method, (3) sample preparation steps, (4) references for collection protocols. In the data generation phase, we considered (5) the pulse sequence(s) applied for data acquisition, (6) references for data acquisition protocols (7) whether the study generated binned data, metabolite concentrations or both, (8) uniform or variable width binning, (9) method used for metabolite profiling, as the chosen method impacts the data produced and data sharing capabilities. In the data analysis phase, we extracted (10) pre-treatment strategies, (11) tests for normality, (12) unsupervised multivariate analyses, (13) supervised multivariate analyses, (14) univariate analyses, (15) multiple testing correction, as it reflects the way the data are packaged for consumption though publication. The compiled studies used a diverse range of procedures in their metabolomics workflow summarised in Figure 3. We discuss the nature of this diversity in the following sections. 

### 3.2. Pre-Analytical Phase 

There were 25 different biospecimens identified in our systematic review, including serum, urine, plasma, tissue, cerebral spinal fluid, follicular fluid, stool, exhaled breath condensate, saliva, aqueous humour, bile, platelets, seminal fluid, sputum, synovial fluid, amniotic fluid, acute promyelocytic blasts, interstitial fluid, mouth wash out, pancreatic juice, peritoneal effusion, red blood cells, tears, tongue swab, and whole blood. The most popular were serum (32.2%), urine (22.1%), and plasma (16.3%). These biofluids are generally minimally invasive and relatively simple to collect. 

### 3.3. Blood Collection

Whole blood from patients is often altered upon collection by the tubes and processing steps that come after. Serum and plasma are the most commonly analysed blood extracts but analysis can be extended to whole blood [181], platelets [103], red blood cells [56], peripheral blood mononuclear cells [182] and dried blood spots [183]. 

Plasma is obtained after the removal of cells and platelets via centrifugation. It is the liquid proportion of blood that contains clotting factors and protein. Plasma is collected in tubes containing an anticoagulant. Overall, 41.2% of plasma studies used ethylenediaminetetraacetic acid (EDTA) tubes, 14.7% used heparin followed by 2.9% using sodium citrate and 41.2% not reporting the nature of the collection tube. The anticoagulant should be chosen carefully as it may inhibit other biological processes and influence the metabolic profile. Heparin inhibits coagulation activator (thrombin) whereas EDTA and citrate chelate divalent metal ions, such as calcium and magnesium, thus inhibiting magnesium-dependent coagulation enzymes [183]. EDTA produces intense peaks in NMR spectra, obscuring signals from choline, dimethylamine, and citrate; and samples may contain endogenous citrate. Therefore, it is recommended to avoid EDTA and sodium citrate anti-coagulants [184]. Conversely, it has also been suggested to avoid heparin as it causes broad peaks in the spectrum complicating lipid quantitation [185]. Furthermore, 38.2% plasma studies reported centrifugation parameters (rotor speed, time, temperature) for processing at collection, 41.2% prior to sample preparation and 14.7% reported at both steps. For each of the biofluids (including urine), there were a diverse range of rotor speeds, the centrifugation time ranged from 5 to 20 min and temperature reported included 4 °C or at room temperature. Not all studies reported all three parameters; a detailed breakdown can be found in the Supplementary Data.

An alternative to plasma that does not require addition of anticoagulants that interfere with resonances of the NMR spectra is serum. Serum consists of similar constituents as plasma with the absence of clotting factors. It is prepared by letting whole blood sit at room temperature for clot formation, usually for 30 min. Only 31.3% of serum studies reported a clot time, which ranged from 15 to 240 min. During clotting time, enzymatic reactions and degradation can still occur. For example, the activation of platelets can release additional compounds such as hypoxanthine, xanthine, and amino acids [186]. Overall, 19.4% of serum studies used tubes containing no additives, 7.5% a gel separator, 4.5% silica-coated tubes and 68.7% did not report the type of tube. Centrifugation parameters were reported for 50.7% serum studies at collection, 23.9% before sample preparation and 7.5% reported at both steps.

### 3.4. Urine Collection

Urine samples can be collected under different conditions. We identified four different urine types; first morning void (41.3%), random urine (6.52%), 24 h urine (4.35%), spot urine (2.17%), and 45.7% did not report the condition of urine collection. First morning void occurs following an overnight fast and is least likely to be affected by daily routine. Random urine can be collected at any time of the day and may induce the most unwanted variability from different collection times and conditions. We considered a sample to be random urine if it was collected after appointments or treatments occurring at unspecified times during the day. In contrast, spot urine is collected at specified times. We have defined second morning void as spot urine, as it is collected under pre-defined conditions without fasting. The 24 h urine is a pooled sample of all voids within a 24 h period [187]. This sample type can average out fluctuations from the circadian cycle. 

Another consideration for urine collection is bacterial contamination. Overall, 21.7% of urine studies reported midstream collection which reduces the risk of collecting a sample contaminated from the urinary tract [188]. Further, removing cells and bacteria from the sample is a standard practice that is achieved with mild centrifugation and/or filtration with a 0.20 μm syringe filter [185]. In all, 63.0% urine studies reported performing centrifugation and/or filtration, with the remainder unknown. Meanwhile, 33% studies reported centrifugation parameters at collection, 43.5% before sample preparation for the thawed sample and 15.2% reported for both steps.

### 3.5. Sample Preparation 

There are additional sample preparation steps that may be included in the workflow to isolate metabolites from macromolecules that remain in the samples after treatment. These steps include ultrafiltration or metabolite extraction. We found that 82.2% of blood (serum and plasma) studies did not perform additional sample preparation steps, 9.5% performed ultrafiltration and 7.9% performed metabolite extraction. Overall, 4.3% of urine studies performed ultrafiltration, and the remainder conducted no preparation. 

Part of sample preparation is converting raw biofluid into an optimal medium that is suitable for the analytical instrument. Deuterated phosphate buffer is often added to the sample prior to NMR data acquisition. Adding buffer maintains a constant pH (physiological pH 7.4) across all the samples which minimises metabolite chemical shift variations and allows for more accurate metabolite identification against library standards. Sodium or potassium phosphate buffer was used in 79.6% studies, 6.1% used deuterated water (D_2_O) only, 2.7% used saline solution with two deuteration levels (100% or 10%) and 0.7% (one urine study) used Chenomx Internal Standard Solution consisting of D_2_O, sodium trimethylsilylpropanesulfonate (DSS) and sodium azide (Table 1). Sodium azide (NaN_3_) can also be added in the buffer to prevent bacterial growth [189], which 30.6% studies had reported. Overall, 68.7% studies reported a chemical shift reference, with trimethylsilylpropanoic acid (TSP) (54.5%) and DSS (10.2%) being the most popular. 

### 3.6. Data Generation Phase

#### 3.6.1. NMR Introduction

NMR is a powerful analytical technique known for its ability to characterise molecular structures and dynamics. The majority of NMR applications take advantage of NMR-active nuclei from isotopes such as ^1^H, ^13^C, ^15^N, ^31^P. ^1^H has 99% natural abundance and is present in most metabolites, including amino acids, sugars and fatty acids making it particularly useful and popular for the identification of known metabolites. A single 1D ^1^H NMR spectrum can capture hundreds to thousands of signals from molecules that may be low or high in molecular weight [191]. There are different NMR experiments with various pulse sequences, which are series of microsecond radio frequency pulses and magnetic gradients that can be manipulated to excite the active nuclei to produce characteristic NMR spectra. 

#### 3.6.2. NMR Experiments

The non-destructive nature of NMR allows multiple NMR experiments to be applied to a single sample. Overall, 69.4% of studies performed a single experiment, 29.4% performed multiple experiments and 0.7% did not report the type of experiment. We identified two main experiments which were the 1D Carr-Purcell-Meibom-Gill (CPMG) experiment with presaturation for solvent suppression and T_2_ relaxation filtering and the 1D nuclear Overhauser enhancement spectroscopy (NOESY) experiment also with presaturation [184]. Overall, 48.3% of studies performed a CPMG experiment, 46.3% performed a NOESY experiment, 9.5% studies sent their samples to the Nightingale Health metabolomics platform for data acquisition and generation [192], 8.8% performed a 2D J-resolved experiment [193], 8.2% performed a diffusion-edited experiment [194] and 8.8% applied other NMR experiments (Supplementary Table S1). 

**Figure 4:**
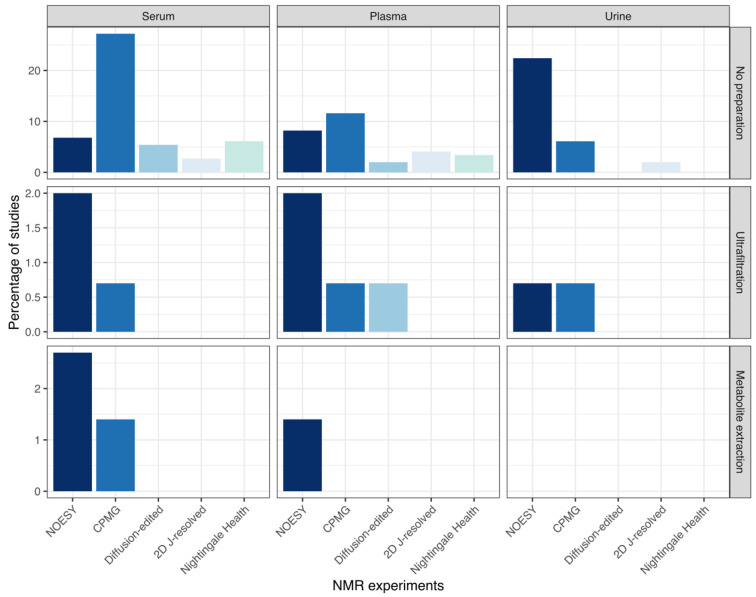
Bar graph showing how data are acquired on NMR. Different NMR experiments and the Nightingale Health platform used to generate NMR data from different sample preparation steps in serum, plasma, and urine studies.

Different NMR pulse sequences can exploit the behaviour of nuclei to produce a characteristic spectrum. The 1D presaturation NOESY pulse sequence suppresses the water signal, without sacrificing the signal intensity of the majority of metabolite peaks, capturing high and low molecular weight compounds [195]. The CPMG experiment attenuates signals that have short transverse relaxation times, such as large proteins and lipoproteins, leaving only slowly relaxing small metabolites and those signals from slow relaxing protons, such as methyl groups of lipid signals in the spectrum. Since serum, plasma and urine consist of different molecular constituents and NMR experiments can filter or select for molecules, their relationship is shown in Figure 4. 

Briefly, Nightingale Health has created a high-throughput and automated NMR metabolomics platform for serum and plasma samples; able to detect lipids, lipoprotein particles and subclasses (LIPO), and low molecular weight metabolites (LMWM) [190,192]. It uses robotics to prepare the blood samples for the LIPO and LMWM spectra, applying the ^1^H NOESY and CPMG pulse sequences, respectively. A manual lipid extraction is performed, and a third lipid spectrum is acquired using ^1^H NOESY pulse sequence. All the spectra are automatically processed (including phase correction, baseline correction, spectrum alignment) and metabolites are automatically identified and quantified using in-house software based on Bayesian modelling [196]. The 2D ^1^H NMR J-resolved experiment separates the scalar coupling and chemical shifts of resonant peaks into two dimensions which spread out overlapping signals, aiding in the identification of metabolites [193]. Diffusion-edited experiments can produce a spectrum only containing metabolites by subtracting the macromolecule spectrum from the whole spectrum based on the diffusion coefficient of the nuclei [194,197].

There were a few research groups that were frequently referenced for sample preparation and data acquisition: Beckonert et al. (2007) [184], Bernini et al. (2011) [185] Dona et al. (2014) [3], and Soininen et al. (2015) [192] which describe the sample preparation and data acquisition parameters used in the Nightingale Health NMR metabolomics platform. Their parameters are described in Table 1. 

#### 3.6.3. Spectral Binning

NMR spectra of biological mixtures yield information-rich data that can be quantified by spectral binning or metabolite profiling where for the latter concentrations are determined. We found 39.5% of studies generated binned data only, 35.4% analysed metabolite concentrations only, and 25.2% investigated both. There was a higher proportion of urine studies (80.4%) compared to blood that generated binning data, with 48.0% also generating metabolite concentrations. More blood studies generated metabolite concentrations only (42.6%), while 32.7% employed binning only and 24.8% for both.

Spectral binning offers a rapid and consistent method in identifying global trends in spectral peak patterns without the initial need for metabolite identification [198]. The simplest method is uniform binning. It divides the spectrum into equal widths (for example 0.01, 0.04 ppm). Software from Bruker including AMIX and AssureNMR, Mnova, Chenomx, MATLAB, ACD/Labs, NMRProcFlow [199], dataChord Spectrum Miner [200], KnowItAll Software (John Wiley & Sons, Inc., Hoboken, NJ, USA) were used to generate uniform binning data (Supplementary Table S1). Due to the small spectral widths (10–12 ppm) of ^1^H NMR spectra, metabolite peaks can overlap and summate therefore a bin may not always contain a single peak corresponding to a single ^1^H moiety of a metabolite. Further, peak-shift problems and the presence of noise can influence downstream analyses. This has prompted the development of various intelligent, adaptive, and dynamic binning algorithms to account for intensity variation and multiple metabolite peaks [201,202,203,204]. The basic concept behind these algorithms is to isolate every peak by determining local maxima and minima, form bin boundaries with variable widths and subsequently remove noise. Out of the studies that generated binned data, 16.3% used variable width bins generated from algorithms or manual integration. 

Bins that show significant differences between groups are identified in the downstream data analysis. Peaks of the metabolites within these bins are assigned based on their chemical shift by referencing to various databases such as the Human Metabolome Database (HMDB), Biological Magnetic Resonance Bank (BMRB), BBIOREFCODE (Bruker) and assignments reported in the literature.

#### 3.6.4. Metabolite Profiling

Metabolite profiling involves fitting the sample spectrum to pure known metabolite spectra. Through this process, metabolites are both identified and quantitated. Fully automated metabolite profiling remains a challenge in the NMR-based metabolomics workflow due to overlapping metabolite signals and slight chemical shift differences from pH, ionic strength, temperature, and biological matrix discrepancies. Many studies resort to manual or semi-automatic deconvolution methods and commercial software which can be slow, subjective, and error prone. 

Our findings suggest that commercial metabolite profiling tools or platforms were preferred over open source. The most frequently used tools or platforms included Chenomx (42.7%), Nightingale Health (15.7%) and Bruker (AMIX, IVDr, PERCH Solutions) (14.4%). To maximise accuracy of automatic fitting and quantitation, samples should be acquired with the same experimental parameters as used for the reference library. Chenomx provides standard operating protocols (SOPs) (Table 1) for sample preparation and their own NMR acquisition parameters. Despite their popularity as a metabolite profiling tool, only one profiling study used the Chenomx SOP (1.1%). Open source web-servers including MAGMET [205], MetaboHunter [206] and Metabominer [207] were each employed once (1.1%) by profiling studies. Various deconvolution algorithms exist [208], however only BATMAN [209] appeared (also once, 1.1%). 

### 3.7. Data Analysis Phase

#### 3.7.1. Data Pre-Treatment

Metabolomic data may be subjected to confounding biological and experimental variations. Therefore, it is necessary to perform pre-treatment such as normalisation, scaling, and transformation to produce a “clean” dataset that make samples (participants) and variables (bin integrals or metabolite concentrations) more comparable and suitable for specific analyses. The application of various pre-treatment methods emphasize different aspects of the data and can profoundly affect biological interpretation [210]. 

We found two main normalisation techniques: 32.0% used total sum; 12.2% used probabilistic quotient normalisation (PQN); and 41.5% did not report a normalization technique. Total sum represents each variable relative to all the number of variables [211]. PQN divides each sample spectrum by a reference spectrum that is representative of the median [212]. The advantage of PQN is that regions of the spectrum are normalised against itself, therefore areas of interest are not influenced by the rest of the spectrum. Both total sum and PQN are global normalisation approaches [213]. Other approaches include referencing the spectra to a single entity that can either be the chemical shift reference or an endogenous metabolite such as creatinine and formate (Supplementary Table S1). 

Unit variance scaling, also known as autoscaling was performed by 30.6% of the studies, 19.3% performed pareto scaling, 3.4% performed mean centring only and 46.7% did not report. Unit variance scaling and pareto scaling initially involves mean centring the data. All the metabolite concentrations therefore fluctuate around zero, accounting for any bias that may favour abundant metabolites. Autoscaling subsequently divides each variable by their standard deviation so that all metabolites are treated with equal importance in downstream analyses and pareto scaling divides the mean-centred data by the square root of the standard deviation to simulate the relative abundance of metabolites from the original dataset in the scaled dataset [210]. 

#### 3.7.2. Multivariate Analyses

Multivariate analysis was performed by 87.1% of the studies. The majority began with unsupervised principal components analysis (PCA), followed by a supervised classification model and the identification of key metabolites through variable importance scores. PCA was a common analysis and was applied by 68.0% of the studies that performed multivariate analysis. PCA is a dimensionality reduction technique which reconstructs high-dimensional data into linear combinations called principal components that preserve the maximum variation observed in the original dataset [214]. The variation can separate the data into different clusters. Being an unsupervised analysis, PCA does this without the knowledge of class labels, which is the attribute of interest that defines a group; in metabolomics studies class labels are often the presence or absence of a disease. 

Projection to Latent Structures Discriminant Analysis (PLS-DA) is a classification machine learning algorithm [215]. Like PCA, it also performs dimensionality reduction by generating linear combinations, however with the knowledge of class labels. Overall, 42.2% of multivariate studies performed PLS-DA, which has two additional variations: orthogonal PLS-DA (OPLS-DA) and sparse PLS-DA (sPLS-DA). The best performing PLS-DA variant is debated in the literature [216]. Both variations are aimed to identify important variables and remove those that do not contribute to the prediction of the class label. OPLS-DA was performed by 48.4% of multivariate studies and 0.5% performed sPLS-DA. The weakness of PLS-DA is that it is prone to overfitting, which means they rely heavily on the initial training dataset for accurate prediction. 

Other unsupervised and supervised machine learning algorithms that appeared in our literature search included random forest (11.7%), hierarchical clustering analysis (5.5%), support vector machine (4.7%), k-means clustering (2.3%), and t-SNE (0.8%). Although outside the scope of this review, many reviews exist that explore the broad array of statistical objectives, misconceptions, and pitfalls implemented [217,218]. At this stage, the studies from our systematic review showed that machine learning models are still at the training stage and are yet to become inferential. 

#### 3.7.3. Univariate Analyses

Univariate analysis was performed by 85.7% of studies. It uses one variable to describe or infer a conclusion on a sample population using hypothesis testing or univariate generalised linear modelling for testing the strength of association between the metabolite and class label. Here, we will mainly discuss how hypothesis testing is used in metabolomic studies. Choosing the correct hypothesis test for the data can yield more reliable results which can be achieved by meeting all assumptions the test relies upon, e.g., normality, independence, and equal variance [219,220]. 

A normal (gaussian) distribution is when observations appear most frequently around the mean value, which can be visualized as a symmetrical bell-shaped plot. To assess for normality, tests reported in our studies included Kolmogorov-Smirnoff, Shapiro–Wilk, D’Agostino-Pearson, and histograms and Q-Q plots for visual inspection. Overall, 90.5% of studies that performed hypothesis testing did not perform a normality test prior. 

Simultaneously performing multiple hypothesis tests for the large number of metabolite features increases the possibility of false positives (Type I error). Significance levels and *p*-values need to be adjusted which is referred as multiple testing correction [221]. Studies reported controlling the false discovery rate inclusive of Benjamini–Hochberg and Benjamini–Yekutieli procedures, and the family-wise error rate including the Bonferroni correction (Supplementary Table S1). Out of the studies that performed hypothesis testing, 36.5% also adjusted for multiple testing. Some may argue that multiple testing correction is not necessary as it may be more detrimental to the research if potential significant metabolites were missed. Considering this, the multiple testing corrections have different thresholds which can be applied based on the exploratory nature of the research question. 

## 4. Discussion

The main aim of this systematic review of NMR-based metabolomics research on human biofluids was to determine how much variation in workflow existed between different studies. Although various tissue, cells and compartmentalised fluids may provide more localised significance and relevance to metabolic perturbations observed in a disease, serum, plasma, and urine accounted for more than 70% of the studies in our literature search. The variation of workflows on these three biofluids (visualised in Figure 3) is quite substantial, especially for serum and plasma. 

Serum and plasma are extracts of whole blood. Previous studies found that the quantitation of metabolites in plasma was more reproducible due to reduced handling [222], however higher concentrations of amino acids were found in serum which may be explained by volume displacement effects [223]. A recent study investigating the impact of collection tubes between serum and plasma revealed that heparin plasma, followed by EDTA plasma had a closer metabolic profile to serum collected in tubes with no-additives [224]. Further, serum separator tubes containing polymeric gel has been shown to alter metabolite levels therefore it is recommended to use additive-free glass or plastic tubes [184]. Another pre-analytical factor that needs to be considered are the centrifugation parameters. The force applied may affect platelet count [225] or cause hemolysis which alter metabolite levels in blood [2]. Various studies have investigated the influence of different centrifugation parameters have on serum, plasma and urine metabolomes and developed optimal protocols for their respective processing [226,227].

The goal of sample collection and sample preparation steps is to preserve the metabolic composition of the sample to ensure an accurate representation of the metabolome at the time of collection. Therefore, SOPs specific for metabolomics studies are crucial as slight variations in sample collection, processing and storage conditions can significantly affect metabolite stability and abundance [183]. However, the issue of having numerous best practices remains and it is up to the researcher to be well-versed and transparent in their decisions. To achieve this, complete reporting of collection method including collection tubes and durations and processing parameters are mandatory for reproducibility and to reduce analytical bias. 

Sample preparation steps are often used to enhance the metabolite peak signals of the NMR spectra. Macromolecules, such as protein and lipids, give rise to intense and broad signals in NMR spectra, obscuring metabolite signals, and the chemical shift reference may bind to protein causing difficulties and errors in metabolite quantification. Ultrafiltration (with 3 kDa or 3.5 kDa cut-off) is the simplest and fastest method for removing macromolecules. However, metabolites can be lost in the filter membrane and protein-bound metabolites are filtered out along with the protein [228]. Liquid-liquid extraction (LLE) is a metabolite extraction method that uses organic solvents to precipitate protein and separate polar metabolites and lipids into hydrophilic and hydrophobic phases, respectively. Using deuterated methanol and chloroform has been shown to prevent further enzymatic activity [229] and yield efficient recovery of metabolites [230]. LLE appears to enhance metabolite peaks compared to ultrafiltration [230], most likely due to the capture of protein-bound metabolites after precipitation. Solvent peaks that are introduced into the NMR sample may be removed via lyophilisation, however this process will remove some of the volatile metabolites. The chemical shift reference is used for spectra alignment and absolute metabolite quantitation. Without a chemical shift reference, spectra can be aligned using isolated metabolite peaks that are unlikely to be affected by pH, however, with this approach metabolites cannot be absolutely quantitated.

The NOESY pulse sequence can consistently generate high-quality spectra with short acquisition time, and it is relatively simple to set up with few optimisation parameters, suitable for the non-spectroscopist. The disadvantage of the NOESY pulse sequence is that the solvent suppression can leave the baseline distorted, or alter signal intensities near the suppressed solvent peak [231]. Distorted baselines will cause difficulties for automatic processing and may introduce inaccurate or subjective corrections. When a sample contains high molecular weight compounds such as protein and lipoproteins, they create broad peaks in the NMR spectrum which may dominate metabolites signals, therefore a CPMG pulse sequence is more ideal for quantitating metabolites in biospecimens with high protein content. Another consideration of using the CPMG pulse sequence is that protein-bounded metabolites will not be observed in the NMR spectrum as they share an effective relaxation time with the protein and are filtered out together with the protein signals. Furthermore, lipids in the sample can obscure upfield metabolite signals. As part of the MSI guidelines [6], all instrument parameters should be fully reported so that experimental procedures can be replicated; all studies except for one followed this requirement either briefly describing the parameters or referencing the protocol followed. 

For the sake of comparing NMR data between separate studies it is most important that sample collection, sample preparation and data generation techniques are as consistent as possible. Urine was shown to have a very consistent workflow of neat collection, no preparation, and NOESY pulse sequence being applied in 71.7% of studies. Serum and plasma were both inconsistent and this was confounded by a lack of reported information on collection tubes used to produce the serum or plasma. Removing the issue of the collection tube from the equation, the most common combination for serum and plasma was no preparation and CPMG pulse sequence (56.4% of blood studies). The Nightingale Health NMR metabolomics platform provides a consistent combination of collection factors; however, the limitation of this platform is that spectra are not provided, so binning analysis cannot be performed. Minimal handling of samples is a sensible way to reduce human error and manipulation of in vivo measurements of blood. Such minimal handling may explain why no sample preparation is the most common method for NMR-based metabolomics.

The generation of metabolite data can be considered one side of the workflow, the other side is the analysis and interpretation of that data. The data analysis workflow and the presentation of data in research papers was highly varied between projects. There are many ways to look at data, but the standardisation of the steps appears to be limited. Beyond variation, there appeared to be minimal justification for the data analysis steps taken by researchers presenting metabolomics data. Prior to the analysis of data, the data needs to be converted from the NMR spectral format to a numerical format. This conversion of data can be performed by profiling metabolite concentrations or by producing bins that quantitate the area under the spectrum. 

Datasets continue to grow and become more complex therefore requiring proper pre-treatment. Specific scaling strategies should be applied for different statistical analyses as the aim of the scaling method should match what the analysis is measuring. There was a large proportion of studies that did not report a normalisation or scaling strategy, we strongly encourage researchers to provide a detailed reasoning for their choice, or lack of pre-treatment. 

Although hypothesis testing procedures are routine tests seen in metabolomics studies, we found that they were misused. Both parametric hypothesis tests, which are tests that meet the conditions of a normal distribution, and non-parametric tests, which are tests for non-normally distributed data, were often conducted without first assessing normality [232]. Parametric tests are more powerful than non-parametric, therefore significant metabolites may be inadvertently deemed important or missed if either parametric or non-parametric tests are applied with incorrect normality assumptions [233]. Once an initial assessment for normality is conducted, non-normal data may be scaled or transformed so that a parametric test can be performed. An alternative is algorithmic modelling or machine learning which uses functions to identify patterns within the data and is measured based on prediction accuracy [234]. It is important to recognise that there are several models that may be applicable to the data [234], therefore it is crucial to describe the biological interpretation in context with the analyses performed, whether it is univariate, multivariate or algorithmic-based. The drawback for machine learning algorithms is that large sample sizes are required for accurate prediction, whereas most sample sizes for metabolomics studies are still relatively small. We believe that the standardisation in data generation for data collation will pave the way for the next advancements of metabolomics and machine learning applications.

While this systematic review aims to provide an update on current methods commonly employed in the NMR metabolomics workflow and point out the extent of their variation between studies, we still expect to see variation in the future as improvements are made to existing methods or new methodologies are developed. The necessity for standardisation is often debated as there are continued advancements made in the NMR metabolomics field such as sample collection devices [235,236], data processing software [237], NMR experiments and pulse sequences [238], improved instrument sensitivity [239,240], and metabolite deconvolution software [241,242,243]. Therefore, when using new methods, we encourage careful considerations in their implementation and detailed reporting.

## 5. Conclusions

Our analysis of metabolite disease studies published in 2019 and 2020 has highlighted that significant variation exists in NMR-based metabolomics data generation and data analysis workflows. The variation in data analysis workflows is expected but more justification of steps taken should be reported. Given that NMR reproducibility is a real strength to using this platform for metabolomics, we recommend using data generation workflows that are consistent within the field while leaning towards minimal sample handling steps. For urine, there is a consistent workflow of neat collection, no preparation and using the NOESY pulse sequence to acquire NMR data (this data generation workflow is likely most suitable for any biofluid that lacks macromolecules). For serum and plasma, there are inconsistencies between studies, but the use of glass tubes without additives for serum and heparin for plasma appear to be the most common; this is followed by no preparation step and using both NOESY and CPMG pulse sequences for acquiring NMR data. Overall, this review can act as a valuable starting resource for any research group wanting to standardise their research and make it suitable for data collation and comparison. 

## Figures and Tables

**Table 1:** Comparison of the final buffer concentrations and pulse sequence combinations for serum/plasma and urine.

Reference	Collection Tube	Sample Prep	Sample: Buffer Ratio	Buffer	% D_2_O	Chemical Shift Reference (mM)	NaN_3_ (mM)	pH	NMR Experiments	Temp (K)
Plasma										
Beckonert [184]	Li-heparin		1:2	103 mM NaCl	6.66				noesy, cpmg, (jres, diff)	310
Bernini [185]	EDTA or citrate		1:1	35 mM Na_2_HPO_4_	10	TSP (27.5)	19	7.4	noesy, cpmg	310
Dona [3]	Li-heparin or EDTA		1:1	37.5 mM NaH_2_PO_4_	10	TSP (2.73)	3.08	7.4	noesy, cpmg, jres	310
Soininen [190]			1:1	37.5 mM Na_2_HPO_4_	10	TSP-d_4_ (2.32)	3.08	7.4	noesy, cpmg	310
Chenomx		Ultra- filtration 3 kDa	9:1	D_2_O	10	DSS-d_6_ (0.5)	1.54		noesy	298
Urine										
Beckonert [184]	0.05% wt/vol NaN_3_		2:1	82.3 mM Na_2_HPO_4_	6.66	TSP (0.33)	1	7.4	noesy	300
Bernini [185]	3 mM NaN_3_		9:1	150 mM K_2_HPO_4_	10	TSP (1.0)		7.4	noesy, jres	300
Dona [3]	0.05% wt/vol NaN_3_		9:1	150 mM KH_2_PO_4_	1	TSP (0.68)	0.2	7.4	noesy, jres	300
Chenomx			9:1	D_2_O	10	DSS-d_6_ (0.5)	1.54		noesy	298

Serum follows the same parameters as plasma minus the collection tube. NMR experiments listed in brackets were optional recommendations. TSP-d_4_: 3-(Trimethylsilyl)propionic-2,2,3,3-d_4_ acid sodium salt. DSS-d_6_: 3-(Trimethylsilyl)-1-propanesulfonic acid-d_6_ sodium salt.

## Data Availability

Compiled dataset in an excel spreadsheet available in Supplementary Information.

## Supplementary Materials

The following supporting information can be downloaded at: https://www.mdpi.com/article/10.3390/metabo12100963/s1, Table S1: Summary table for serum, plasma, and urine.

## References

[1] Wishart D.S. (2016). Emerging applications of metabolomics in drug discovery and precision medicine. Nat. Rev. Drug Discov..

[2] Gonzalez-Dominguez R., Gonzalez-Dominguez A., Sayago A., Fernandez-Recamales A. (2020). Recommendations and Best Practices for Standardizing the Pre-Analytical Processing of Blood and Urine Samples in Metabolomics. Metabolites.

[3] Dona A.C., Jimenez B., Schafer H., Humpfer E., Spraul M., Lewis M.R., Pearce J.T., Holmes E., Lindon J.C., Nicholson J.K. (2014). Precision high-throughput proton NMR spectroscopy of human urine, serum, and plasma for large-scale metabolic phenotyping. Anal. Chem..

[4] Lu W., Su X., Klein M.S., Lewis I.A., Fiehn O., Rabinowitz J.D. (2017). Metabolite Measurement: Pitfalls to Avoid and Practices to Follow. Annu. Rev. Biochem..

[5] Vignoli A., Ghini V., Meoni G., Licari C., Takis P.G., Tenori L., Turano P., Luchinat C. (2019). High-Throughput Metabolomics by 1D NMR. Angew. Chem. Int. Ed. Engl..

[6] Sumner L.W., Amberg A., Barrett D., Beale M.H., Beger R., Daykin C.A., Fan T.W., Fiehn O., Goodacre R., Griffin J.L. (2007). Proposed minimum reporting standards for chemical analysis Chemical Analysis Working Group (CAWG) Metabolomics Standards Initiative (MSI). Metabolomics.

[7] Members M.S.I.B., Sansone S.A., Fan T., Goodacre R., Griffin J.L., Hardy N.W., Kaddurah-Daouk R., Kristal B.S., Lindon J., Mendes P. (2007). The metabolomics standards initiative. Nat. Biotechnol..

[8] Spicer R.A., Salek R., Steinbeck C. (2017). Compliance with minimum information guidelines in public metabolomics repositories. Sci. Data.

[9] R Development Core Team (2020). R: A Language and Environment for Statistical Computing.

[10] Kumar U., Sharma S., Durgappa M., Gupta N., Raj R., Kumar A., Sharma P.N., Krishna V.P., Kumar R.V., Guleria A. (2021). Serum Metabolic Disturbances Associated with Acute-on-chronic Liver Failure in Patients with Underlying Alcoholic Liver Diseases: An Elaborative NMR-based Metabolomics Study. J. Pharm. Bioallied Sci..

[11] Rocca M.S., Vignoli A., Tenori L., Ghezzi M., De Rocco Ponce M., Vatsellas G., Thanos D., Padrini R., Foresta C., De Toni L. (2020). Evaluation of Serum/Urine Genomic and Metabolomic Profiles to Improve the Adherence to Sildenafil Therapy in Patients with Erectile Dysfunction. Front. Pharmacol..

[12] Izquierdo-Garcia J.L., Comella-Del-Barrio P., Campos-Olivas R., Villar-Hernández R., Prat-Aymerich C., De Souza-Galvão M.L., Jiménez-Fuentes M.A., Ruiz-Manzano J., Stojanovic Z., González A. (2020). Discovery and validation of an NMR-based metabolomic profile in urine as TB biomarker. Sci. Rep..

[13] Citterio F., Romano F., Meoni G., Iaderosa G., Grossi S., Sobrero A., Dego F., Corana M., Berta G.N., Tenori L. (2020). Changes in the Salivary Metabolic Profile of Generalized Periodontitis Patients after Non-surgical Periodontal Therapy: A Metabolomic Analysis Using Nuclear Magnetic Resonance Spectroscopy. J. Clin. Med..

[14] Yang F., Li Q., Xiang J., Zhang H., Sun H., Ruan G., Tang Y. (2021). NMR-based plasma metabolomics of adult B-cell acute lymphoblastic leukemia. Mol. Omics.

[15] Värri M., Niskanen L., Tuomainen T.P., Honkanen R., Kröger H., Tuppurainen M.T. (2020). Metabolite Profiling of Osteoporosis and Atherosclerosis in Postmenopausal Women: A Cross-Sectional Study. Vasc. Health Risk Manag..

[16] Ghini V., Laera L., Fantechi B., Monte F.D., Benelli M., McCartney A., Leonardo T., Luchinat C., Pozzessere D. (2020). Metabolomics to Assess Response to Immune Checkpoint Inhibitors in Patients with Non-Small-Cell Lung Cancer. Cancers.

[17] Ren Y., Chen Z.Z., Sun X.L., Duan H.J., Tian J.S., Wang J.Y., Yang H. (2021). Metabolomic analysis to detect urinary molecular changes associated with bipolar depression. Neurosci. Lett..

[18] Paris D., Palomba L., Mirra V., Borrelli M., Corcione A., Santamaria F., Maniscalco M., Motta A. (2020). NMR Profiling of Exhaled Breath Condensate Defines Different Metabolic Phenotypes of Non-Cystic Fibrosis Bronchiectasis. Int. J. Mol. Sci..

[19] Nizioł J., Ossoliński K., Tripet B.P., Copié V., Arendowski A., Ruman T. (2021). Nuclear magnetic resonance and surface-assisted laser desorption/ionization mass spectrometry-based metabolome profiling of urine samples from kidney cancer patients. J. Pharm. Biomed. Anal..

[20] Chachaj A., Matkowski R., Gröbner G., Szuba A., Dudka I. (2020). Metabolomics of Interstitial Fluid, Plasma and Urine in Patients with Arterial Hypertension: New Insights into the Underlying Mechanisms. Diagnostics.

[21] Quintero Escobar M., Costa T., Martins L.G., Costa S.S., vanHelvoort Lengert A., Boldrini É., da Silva S.R.M., Lopes L.F., Vidal D.O., Krepischi A.C.V. (2020). Insights in Osteosarcoma by Proton Nuclear Magnetic Resonance Serum Metabonomics. Front. Oncol..

[22] Yilmaz A., Ustun I., Ugur Z., Akyol S., Hu W.T., Fiandaca M.S., Mapstone M., Federoff H., Maddens M., Graham S.F. (2020). A Community-Based Study Identifying Metabolic Biomarkers of Mild Cognitive Impairment and Alzheimer’s Disease Using Artificial Intelligence and Machine Learning. J. Alzheimers Dis..

[23] Castaldo G., Pagano I., Grimaldi M., Marino C., Molettieri P., Santoro A., Stillitano I., Romano R., Montoro P., D’Ursi A.M. (2021). Effect of Very-Low-Calorie Ketogenic Diet on Psoriasis Patients: A Nuclear Magnetic Resonance-Based Metabolomic Study. J. Proteome Res..

[24] Fraser D.D., Slessarev M., Martin C.M., Daley M., Patel M.A., Miller M.R., Patterson E.K., O’Gorman D.B., Gill S.E., Wishart D.S. (2020). Metabolomics Profiling of Critically Ill Coronavirus Disease 2019 Patients: Identification of Diagnostic and Prognostic Biomarkers. Crit. Care Explor..

[25] Sahni S., Pandya A.R., Hadden W.J., Nahm C.B., Maloney S., Cook V., Toft J.A., Wilkinson-White L., Gill A.J., Samra J.S. (2021). A unique urinary metabolomic signature for the detection of pancreatic ductal adenocarcinoma. Int. J. Cancer.

[26] Herrala M., Mikkonen J.J.W., Pesonen P., Lappalainen R., Tjäderhane L., Niemelä R.K., Seitsalo H., Salo T., Myllymaa S., Kullaa A.M. (2020). Variability of salivary metabolite levels in patients with Sjögren’s syndrome. J. Oral Sci..

[27] Haak B.W., Westendorp W.F., van Engelen T.S.R., Brands X., Brouwer M.C., Vermeij J.D., Hugenholtz F., Verhoeven A., Derks R.J., Giera M. (2021). Disruptions of Anaerobic Gut Bacteria Are Associated with Stroke and Post-stroke Infection: A Prospective Case-Control Study. Transl. Stroke Res..

[28] Kwon H.N., Lee H., Park J.W., Kim Y.H., Park S., Kim J.J. (2020). Screening for Early Gastric Cancer Using a Noninvasive Urine Metabolomics Approach. Cancers.

[29] Maignien C., Santulli P., Kateb F., Caradeuc C., Marcellin L., Pocate-Cheriet K., Bourdon M., Chouzenoux S., Batteux F., Bertho G. (2020). Endometriosis phenotypes are associated with specific serum metabolic profiles determined by proton-nuclear magnetic resonance. Reprod. Biomed. Online.

[30] Lins Neto MÁ F., Verdi G.M.X., Veras A.O., Veras M.O., Caetano L.C., Ursulino J.S. (2020). Use of metabolomics to the diagnosis of inflammatory bowel disease. Arq. Gastroenterol..

[31] Piras C., Pibiri M., Leoni V.P., Balsamo A., Tronci L., Arisci N., Mariotti S., Atzori L. (2021). Analysis of metabolomics profile in hypothyroid patients before and after thyroid hormone replacement. J. Endocrinol. Investig..

[32] Barbosa Breda J., Croitor Sava A., Himmelreich U., Somers A., Matthys C., Rocha Sousa A., Vandewalle E., Stalmans I. (2020). Metabolomic profiling of aqueous humor from glaucoma patients—The metabolomics in surgical ophthalmological patients (MISO) study. Exp. Eye Res..

[33] Huhtala M.S., Tertti K., Rönnemaa T. (2020). Serum lipids and their association with birth weight in metformin and insulin treated patients with gestational diabetes. Diabetes Res. Clin. Pract..

[34] Signoriello E., Iardino P., Casertano S., De Lucia D., Pucciarelli A., Puoti G., Chiosi E., Lus G. (2020). 12-months prospective Pentraxin-3 and metabolomic evaluation in multiple sclerosis patients treated with glatiramer acetate. J. Neuroimmunol..

[35] Gómez-Cebrián N., García-Flores M., Rubio-Briones J., López-Guerrero J.A., Pineda-Lucena A., Puchades-Carrasco L. (2020). Targeted Metabolomics Analyses Reveal Specific Metabolic Alterations in High-Grade Prostate Cancer Patients. J. Proteome Res..

[36] Wang S., Wen S., Guo P., Liu H., Feng J., Huang H. (2020). Understanding metabolomic characteristics of pancreatic ductal adenocarcinoma by HR-MAS NMR detection of pancreatic tissues. J. Pharm. Biomed. Anal..

[37] Kimhofer T., Lodge S., Whiley L., Gray N., Loo R.L., Lawler N.G., Nitschke P., Bong S.H., Morrison D.L., Begum S. (2020). Integrative Modeling of Quantitative Plasma Lipoprotein, Metabolic, and Amino Acid Data Reveals a Multiorgan Pathological Signature of SARS-CoV-2 Infection. J. Proteome Res..

[38] Kumari S., Kumaran S.S., Goyal V., Sharma R.K., Sinha N., Dwivedi S.N., Srivastava A.K., Jagannathan N.R. (2020). Identification of potential urine biomarkers in idiopathic parkinson’s disease using NMR. Clin. Chim. Acta.

[39] Gupta L., Guleria A., Rawat A., Kumar D., Aggarwal A. (2021). NMR-based clinical metabolomics revealed distinctive serum metabolic profiles in patients with spondyloarthritis. Magn. Reson. Chem..

[40] Rodríguez-Carrio J., Alperi-López M., López P., Pérez-Álvarez Á.I., Gil-Serret M., Amigó N., Ulloa C., Benavente L., Ballina-García F.J., Suárez A. (2020). GlycA Levels during the Earliest Stages of Rheumatoid Arthritis: Potential Use as a Biomarker of Subclinical Cardiovascular Disease. J. Clin. Med..

[41] Pauzi F.A., Sahathevan S., Khor B.H., Narayanan S.S., Zakaria N.F., Abas F., Karupaiah T., Daud Z.A.M. (2020). Exploring Metabolic Signature of Protein Energy Wasting in Hemodialysis Patients. Metabolites.

[42] Hao D., Sengupta A., Ding K., Ubeydullah E.R., Krishnaiah S., Leighl N.B., Shepherd F.A., Seymour L., Weljie A. (2020). Metabolites as Prognostic Markers for Metastatic Non-Small Cell Lung Cancer (NSCLC) Patients Treated with First-Line Platinum-Doublet Chemotherapy. Cancers.

[43] Prokić I., Lahousse L., de Vries M., Liu J., Kalaoja M., Vonk J.M., van der Plaat D.A., van Diemen C.C., van der Spek A., Zhernakova A. (2020). A cross-omics integrative study of metabolic signatures of chronic obstructive pulmonary disease. BMC Pulm. Med..

[44] Benítez Del Castillo J.M., Pinazo-Duran M.D., Sanz-González S.M., Muñoz-Hernández A.M., Garcia-Medina J.J., Zanón-Moreno V. (2021). Tear 1H Nuclear Magnetic Resonance-Based Metabolomics Application to the Molecular Diagnosis of Aqueous Tear Deficiency and Meibomian Gland Dysfunction. Ophthalmic Res..

[45] Nizioł J., Ossoliński K., Tripet B.P., Copié V., Arendowski A., Ruman T. (2020). Nuclear magnetic resonance and surface-assisted laser desorption/ionization mass spectrometry-based serum metabolomics of kidney cancer. Anal. BioAnal. Chem..

[46] Liang K.H., Cheng M.L., Lo C.J., Lin Y.H., Lai M.W., Lin W.R., Yeh C.T. (2020). Plasma phenylalanine and glutamine concentrations correlate with subsequent hepatocellular carcinoma occurrence in liver cirrhosis patients: An exploratory study. Sci. Rep..

[47] Lalwani A.M., Yilmaz A., Bisgin H., Ugur Z., Akyol S., Graham S.F. (2020). The Biochemical Profile of Post-Mortem Brain from People Who Suffered from Epilepsy Reveals Novel Insights into the Etiopathogenesis of the Disease. Metabolites.

[48] Maltesen R.G., Wimmer R., Rasmussen B.S. (2020). A longitudinal serum NMR-based metabolomics dataset of ischemia-reperfusion injury in adult cardiac surgery. Sci. Data.

[49] Urman J.M., Herranz J.M., Uriarte I., Rullán M., Oyón D., González B., Fernandez-Urién I., Carrascosa J., Bolado F., Zabalza L. (2020). Pilot Multi-Omic Analysis of Human Bile from Benign and Malignant Biliary Strictures: A Machine-Learning Approach. Cancers.

[50] Zennaro L., Nicolè L., Vanzani P., Cappello F., Fassina A. (2020). ^1^H-NMR spectroscopy metabonomics of reactive, ovarian carcinoma and hepatocellular carcinoma ascites. Pleura Peritoneum.

[51] Acar İ.E., Lores-Motta L., Colijn J.M., Meester-Smoor M.A., Verzijden T., Cougnard-Gregoire A., Ajana S., Merle B.M.J., de Breuk A., Heesterbeek T.J. (2020). Integrating Metabolomics, Genomics, and Disease Pathways in Age-Related Macular Degeneration: The EYE-RISK Consortium. Ophthalmology.

[52] Xie J., Chen C., Hou L.J., Zhou C.J., Fang L., Chen J.J. (2020). Dual Metabolomic Platforms Identified a Novel Urinary Metabolite Signature for Hepatitis B Virus-Infected Patients with Depression. Diabetes Metab. Syndr. Obes..

[53] Li Y., Wang Y., Zhuang Y., Zhang P., Chen S., Asakawa T., Gao B. (2020). Serum Metabolomic Profiles Associated With Untreated Metabolic Syndrome Patients in the Chinese Population. Clin. Transl. Sci..

[54] Lima A.R., Pinto J., Barros-Silva D., Jerónimo C., Henrique R., Bastos M.L., Carvalho M., Guedes Pinho P. (2020). New findings on urinary prostate cancer metabolome through combined GC-MS and ^1^H NMR analytical platforms. Metabolomics.

[55] Lee C.W., Yu M.C., Lin G., Chiu J.C., Chiang M.H., Sung C.M., Hsieh Y.C., Kuo T., Lin C.Y., Tsai H.I. (2020). Serum metabolites may be useful markers to assess vascular invasion and identify normal alpha-fetoprotein in hepatocellular carcinoma undergoing liver resection: A pilot study. World J. Surg. Oncol..

[56] Palomino-Schätzlein M., Lamas-Domingo R., Ciudin A., Gutiérrez-Carcedo P., Marés R., Aparicio-Gómez C., Hernández C., Simó R., Herance J.R. (2020). A Translational In Vivo and In Vitro Metabolomic Study Reveals Altered Metabolic Pathways in Red Blood Cells of Type 2 Diabetes. J. Clin. Med..

[57] Metere A., Graves C.E., Chirico M., Caramujo M.J., Pisanu M.E., Iorio E. (2020). Metabolomic Reprogramming Detected by ^1^H-NMR Spectroscopy in Human Thyroid Cancer Tissues. Biology.

[58] Castiglione Morelli M.A., Iuliano A., Schettini S.C.A., Petruzzi D., Ferri A., Colucci P., Viggiani L., Ostuni A. (2020). Metabolic changes in follicular fluids of patients treated with recombinant versus urinary human chorionic gonadotropin for triggering ovulation in assisted reproductive technologies: A metabolomics pilot study. Arch. Gynecol. Obstet..

[59] Capolongo G., Zacchia M., Beneduci A., Costantini S., Cinque P., Spasiano A., De Luca G., Di Pietro M.E., Ricchi P., Trepiccione F. (2020). Urinary Metabolic Profile of Patients with Transfusion-Dependent β-Thalassemia Major Undergoing Deferasirox Therapy. Kidney Blood Press. Res..

[60] Dudka I., Thysell E., Lundquist K., Antti H., Iglesias-Gato D., Flores-Morales A., Bergh A., Wikström P., Gröbner G. (2020). Comprehensive metabolomics analysis of prostate cancer tissue in relation to tumor aggressiveness and TMPRSS2-ERG fusion status. BMC Cancer.

[61] Lunde S., Nguyen H.T., Petersen K.K., Arendt-Nielsen L., Krarup H.B., Søgaard-Andersen E. (2020). Chronic Postoperative Pain After Hysterectomy for Endometrial Cancer: A Metabolic Profiling Study. Mol. Pain.

[62] Malo A.I., Rull A., Girona J., Domingo P., Fuertes-Martín R., Amigó N., Rodríguez-Borjabad C., Martínez-Micaelo N., Leal M., Peraire J. (2020). Glycoprotein Profile Assessed by ^1^H-NMR as a Global Inflammation Marker in Patients with HIV Infection. A Prospective Study. J. Clin. Med..

[63] Andreas N.J., Basu Roy R., Gomez-Romero M., Horneffer-van der Sluis V., Lewis M.R., Camuzeaux S.S.M., Jiménez B., Posma J.M., Tientcheu L., Egere U. (2020). Performance of metabonomic serum analysis for diagnostics in paediatric tuberculosis. Sci. Rep..

[64] Ioannou G.N., Nagana Gowda G.A., Djukovic D., Raftery D. (2020). Distinguishing NASH Histological Severity Using a Multiplatform Metabolomics Approach. Metabolites.

[65] Nong Q., Zhang C., Liu Q., Xie R., Dong M. (2020). Effect of daunorubicin on acute promyelocytic leukemia cells using nuclear magnetic resonance spectroscopy-based metabolomics. Environ. Toxicol. Pharmacol..

[66] Ganguly S., Kumar U., Gupta N., Guleria A., Majumdar S., Phatak S., Chaurasia S., Kumar S., Aggarwal A., Kumar D. (2020). Nuclear magnetic resonance-based targeted profiling of urinary acetate and citrate following cyclophosphamide therapy in patients with lupus nephritis. Lupus.

[67] McCann M.R., McHugh C.E., Kirby M., Jennaro T.S., Jones A.E., Stringer K.A., Puskarich M.A. (2020). A Multivariate Metabolomics Method for Estimating Platelet Mitochondrial Oxygen Consumption Rates in Patients with Sepsis. Metabolites.

[68] Murgia F., Lorefice L., Poddighe S., Fenu G., Secci M.A., Marrosu M.G., Cocco E., Atzori L. (2020). Multi-Platform Characterization of Cerebrospinal Fluid and Serum Metabolome of Patients Affected by Relapsing-Remitting and Primary Progressive Multiple Sclerosis. J. Clin. Med..

[69] Tsai C.K., Lin C.Y., Kang C.J., Liao C.T., Wang W.L., Chiang M.H., Yen T.C., Lin G. (2020). Nuclear Magnetic Resonance Metabolomics Biomarkers for Identifying High Risk Patients with Extranodal Extension in Oral Squamous Cell Carcinoma. J. Clin. Med..

[70] Li L., Zhang M., Men Y., Wang W., Zhang W. (2020). Heavy metals interfere with plasma metabolites, including lipids and amino acids, in patients with breast cancer. Oncol. Lett..

[71] Insenser M., Moncayo S., Martínez-García M., Fernández-Durán E., Samino S., Álvarez-Blasco F., Luque-Ramírez M., Escobar-Morreale H.F. (2020). 2D Diffusion-Ordered ^1^H-NMR Spectroscopy Lipidomic Profiling after Oral Single Macronutrient Loads: Influence of Obesity, Sex, and Female Androgen Excess. Mol. Nutr. Food Res..

[72] Kalantari S., Chashmniam S., Nafar M., Zakeri Z., Parvin M. (2019). Metabolomics approach reveals urine biomarkers and pathways associated with the pathogenesis of lupus nephritis. Iran. J. Basic Med. Sci..

[73] Lee J.S., Kim S.Y., Chun Y.S., Chun Y.J., Shin S.Y., Choi C.H., Choi H.K. (2020). Characteristics of fecal metabolic profiles in patients with irritable bowel syndrome with predominant diarrhea investigated using ^1^H-NMR coupled with multivariate statistical analysis. Neurogastroenterol. Motil..

[74] Vignoli A., Paciotti S., Tenori L., Eusebi P., Biscetti L., Chiasserini D., Scheltens P., Turano P., Teunissen C., Luchinat C. (2020). Fingerprinting Alzheimer’s Disease by ^1^H Nuclear Magnetic Resonance Spectroscopy of Cerebrospinal Fluid. J. Proteome Res..

[75] Merolle L., Marraccini C., Latorrata A., Quartieri E., Farioli D., Scarano L., Fasano T., Bergamini S., Bellei E., Monari E. (2020). Heparin-induced lipoprotein precipitation apheresis in dyslipidemic patients: A multiparametric assessment. J. Clin. Apher..

[76] Souto-Carneiro M., Tóth L., Behnisch R., Urbach K., Klika K.D., Carvalho R.A., Lorenz H.M. (2020). Differences in the serum metabolome and lipidome identify potential biomarkers for seronegative rheumatoid arthritis versus psoriatic arthritis. Ann. Rheum. Dis..

[77] Jaurila H., Koivukangas V., Koskela M., Gäddnäs F., Myllymaa S., Kullaa A., Salo T., Ala-Kokko T.I. (2020). ^1^H NMR Based Metabolomics in Human Sepsis and Healthy Serum. Metabolites.

[78] Liu F., Ruze A., Liu W., Xiong K., Yiming A. (2020). Metabonomic profiling of blood plasma from erectile dysfunction patients using 1H nuclear magnetic resonance spectroscopy. Acta Biochim. Biophys. Sin. (Shanghai).

[79] Gupta A., Bansal N., Mitash N., Kumar D., Kumar M., Sankhwar S.N., Mandhani A., Singh U.P. (2020). NMR-derived targeted serum metabolic biomarkers appraisal of bladder cancer: A pre- and post-operative evaluation. J. Pharm. Biomed. Anal..

[80] Vroegindewey M.M., van den Berg V.J., Oemrawsingh R.M., Kardys I., Asselbergs F.W., van der Harst P., Kietselaer B., Lenderink T., Akkerhuis K.M., Boersma E. (2020). High-frequency metabolite profiling and the incidence of recurrent cardiac events in patients with post-acute coronary syndrome. Biomarkers.

[81] Muhle-Goll C., Eisenmann P., Luy B., Kölker S., Tönshoff B., Fichtner A., Westhoff J.H. (2020). Urinary NMR Profiling in Pediatric Acute Kidney Injury-A Pilot Study. Int. J. Mol. Sci..

[82] Murgia F., Corda V., Serrenti M., Usai V., Santoru M.L., Hurt K.J., Passaretti M., Monni M.C., Atzori L., Monni G. (2020). Seminal Fluid Metabolomic Markers of Oligozoospermic Infertility in Humans. Metabolites.

[83] Cortese N., Capretti G., Barbagallo M., Rigamonti A., Takis P.G., Castino G.F., Vignali D., Maggi G., Gavazzi F., Ridolfi C. (2020). Metabolome of Pancreatic Juice Delineates Distinct Clinical Profiles of Pancreatic Cancer and Reveals a Link between Glucose Metabolism and PD-1(+) Cells. Cancer Immunol. Res..

[84] Jääskeläinen O., Hall A., Tiainen M., van Gils M., Lötjönen J., Kangas A.J., Helisalmi S., Pikkarainen M., Hallikainen M., Koivisto A. (2020). Metabolic Profiles Help Discriminate Mild Cognitive Impairment from Dementia Stage in Alzheimer’s Disease. J. Alzheimers Dis..

[85] Men Y., Li L., Zhang F., Kong X., Zhang W., Hao C., Wang G. (2020). Evaluation of heavy metals and metabolites in the urine of patients with breast cancer. Oncol. Lett..

[86] West K.A., Kanu C., Maric T., McDonald J.A.K., Nicholson J.K., Li J.V., Johnson M.R., Holmes E., Savvidou M.D. (2020). Longitudinal metabolic and gut bacterial profiling of pregnant women with previous bariatric surgery. Gut.

[87] Casadei-Gardini A., Del Coco L., Marisi G., Conti F., Rovesti G., Ulivi P., Canale M., Frassineti G.L., Foschi F.G., Longo S. (2020). ^1^H-NMR Based Serum Metabolomics Highlights Different Specific Biomarkers between Early and Advanced Hepatocellular Carcinoma Stages. Cancers.

[88] Dogan B., Karaer A., Tuncay G., Tecellioglu N., Mumcu A. (2020). High-resolution ^1^H-NMR spectroscopy indicates variations in metabolomics profile of follicular fluid from women with advanced maternal age. J. Assist. Reprod. Genet..

[89] Gooding J.R., Agrawal S., McRitchie S., Acuff Z., Merchant M.L., Klein J.B., Smoyer W.E., Sumner S.J. (2020). Predicting and Defining Steroid Resistance in Pediatric Nephrotic Syndrome Using Plasma Metabolomics. Kidney Int. Rep..

[90] Hsu W.H., Wang S.J., Chao Y.M., Chen C.J., Wang Y.F., Fuh J.L., Chen S.P., Lin Y.L. (2020). Urine metabolomics signatures in reversible cerebral vasoconstriction syndrome. Cephalalgia.

[91] Vignoli A., Tenori L., Giusti B., Valente S., Carrabba N., Balzi D., Barchielli A., Marchionni N., Gensini G.F., Marcucci R. (2020). Differential Network Analysis Reveals Metabolic Determinants Associated with Mortality in Acute Myocardial Infarction Patients and Suggests Potential Mechanisms Underlying Different Clinical Scores Used To Predict Death. J. Proteome Res..

[92] Kumar U., Jain A., Guleria A., Kumar R.V., Misra D.P., Goel R., Danda D., Misra R., Kumar D. (2020). Circulatory Glutamine/Glucose ratio for evaluating disease activity in Takayasu arteritis: A NMR based serum metabolomics study. J. Pharm. Biomed. Anal..

[93] Banoei M.M., Iupe I., Bazaz R.D., Campos M., Vogel H.J., Winston B.W., Mirsaeidi M. (2019). Metabolomic and metallomic profile differences between Veterans and Civilians with Pulmonary Sarcoidosis. Sci. Rep..

[94] Cardner M., Yalcinkaya M., Goetze S., Luca E., Balaz M., Hunjadi M., Hartung J., Shemet A., Kränkel N., Radosavljevic S. (2020). Structure-function relationships of HDL in diabetes and coronary heart disease. JCI Insight.

[95] Righi V., Cavallini N., Valentini A., Pinna G., Pavesi G., Rossi M.C., Puzzolante A., Mucci A., Cocchi M. (2020). A metabolomic data fusion approach to support gliomas grading. NMR Biomed..

[96] Gilany K., Mohamadkhani A., Chashmniam S., Shahnazari P., Amini M., Arjmand B., Malekzadeh R., Nobakht Motlagh Ghoochani B.F. (2019). Metabolomics analysis of the saliva in patients with chronic hepatitis B using nuclear magnetic resonance: A pilot study. Iran. J. Basic Med. Sci..

[97] Liu Y.Y., Yang Z.X., Ma L.M., Wen X.Q., Ji H.L., Li K. (2019). ^1^H-NMR spectroscopy identifies potential biomarkers in serum metabolomic signatures for early stage esophageal squamous cell carcinoma. PeerJ.

[98] Erasmus E., Mason S., van Reenen M., Steffens F.E., Vorster B.C., Reinecke C.J. (2019). A laboratory approach for characterizing chronic fatigue: What does metabolomics tell us?. Metabolomics.

[99] Frick M.A., Barba I., Fenoy-Alejandre M., López-López P., Baquero-Artigao F., Rodríguez-Molino P., Noguera-Julian A., Nicolás-López M., de la Fuente-Juárez A., Codina-Grau M.G. (2019). ^1^H-NMR Urinary Metabolic Profile, A Promising Tool for the Management of Infants with Human Cytomegalovirus-Infection. Metabolites.

[100] Zheng H., Dong B., Ning J., Shao X., Zhao L., Jiang Q., Ji H., Cai A., Xue W., Gao H. (2020). NMR-based metabolomics analysis identifies discriminatory metabolic disturbances in tissue and biofluid samples for progressive prostate cancer. Clin. Chim. Acta.

[101] Silva C.L., Olival A., Perestrelo R., Silva P., Tomás H., Câmara J.S. (2019). Untargeted Urinary ^1^H NMR-Based Metabolomic Pattern as a Potential Platform in Breast Cancer Detection. Metabolites.

[102] Kevat A.C., Carzino R., Vidmar S., Ranganathan S. (2020). Glycoprotein A as a biomarker of pulmonary infection and inflammation in children with cystic fibrosis. Pediatr. Pulmonol..

[103] Chiang J.Y., Lee S.H., Chen Y.C., Wu C.K., Chuang J.Y., Lo S.C., Yeh H.M., Yeh S.S., Hsu C.A., Lin B.B. (2019). Metabolomic Analysis of Platelets of Patients With Aspirin Non-Response. Front. Pharmacol..

[104] Yeo T., Probert F., Jurynczyk M., Sealey M., Cavey A., Claridge T.D.W., Woodhall M., Waters P., Leite M.I., Anthony D.C. (2019). Classifying the antibody-negative NMO syndromes: Clinical, imaging, and metabolomic modeling. Neurol. Neuroimmunol. Neuroinflamm..

[105] Ahmed N., Kidane B., Wang L., Qing G., Tan L., Buduhan G., Srinathan S., Aliani M. (2019). Non-invasive exploration of metabolic profile of lung cancer with Magnetic Resonance Spectroscopy and Mass Spectrometry. Contemp. Clin. Trials Commun..

[106] D’Amato M., Paris D., Molino A., Cuomo P., Fulgione A., Sorrentino N., Palomba L., Maniscalco M., Motta A. (2019). The Immune-Modulator Pidotimod Affects the Metabolic Profile of Exhaled Breath Condensate in Bronchiectatic Patients: A Metabolomics Pilot Study. Front. Pharmacol..

[107] Vignoli A., Santini G., Tenori L., Macis G., Mores N., Macagno F., Pagano F., Higenbottam T., Luchinat C., Montuschi P. (2020). NMR-Based Metabolomics for the Assessment of Inhaled Pharmacotherapy in Chronic Obstructive Pulmonary Disease Patients. J. Proteome Res..

[108] Mehrparvar B., Chashmniam S., Nobakht F., Amini M., Javidi A., Minai-Tehrani A., Arjmand B., Gilany K. (2020). Metabolic profiling of seminal plasma from teratozoospermia patients. J. Pharm. Biomed. Anal..

[109] An J.N., Hyeon J.S., Jung Y., Choi Y.W., Kim J.H., Yang S.H., Oh S., Kwon S., Lee S.H., Cho J.H. (2019). Urinary myo-inositol is associated with the clinical outcome in focal segmental glomerulosclerosis. Sci. Rep..

[110] Wang W., Liu X., Wu J., Kang X., Xie Q., Sheng J., Xu W., Liu D., Zheng W. (2019). Plasma metabolite profiling reveals potential biomarkers of giant cell tumor of bone by using NMR-based metabolic profiles: A cross-sectional study. Medicine (Baltimore).

[111] Zhou Q., Zhang L.Y., Xie C., Zhang M.L., Wang Y.J., Liu G.H. (2020). Metabolomics as a potential method for predicting thyroid malignancy in children and adolescents. Pediatr. Surg. Int..

[112] Tasic L., Larcerda A.L.T., Pontes J.G.M., da Costa T., Nani J.V., Martins L.G., Santos L.A., Nunes M.F.Q., Adelino M.P.M., Pedrini M. (2019). Peripheral biomarkers allow differential diagnosis between schizophrenia and bipolar disorder. J. Psychiatr. Res..

[113] Diao W., Labaki W.W., Han M.K., Yeomans L., Sun Y., Smiley Z., Kim J.H., McHugh C., Xiang P., Shen N. (2019). Disruption of histidine and energy homeostasis in chronic obstructive pulmonary disease. Int. J. Chron. Obstruct. Pulmon. Dis..

[114] Jääskeläinen O., Solje E., Hall A., Katisko K., Korhonen V., Tiainen M., Kangas A.J., Helisalmi S., Pikkarainen M., Koivisto A. (2019). Low Serum High-Density Lipoprotein Cholesterol Levels Associate with the C9orf72 Repeat Expansion in Frontotemporal Lobar Degeneration Patients. J. Alzheimers Dis..

[115] Seow W.J., Shu X.O., Nicholson J.K., Holmes E., Walker D.I., Hu W., Cai Q., Gao Y.T., Xiang Y.B., Moore S.C. (2019). Association of Untargeted Urinary Metabolomics and Lung Cancer Risk Among Never-Smoking Women in China. JAMA Netw. Open.

[116] Bund C., Lhermitte B., Cicek A.E., Ruhland E., Proust F., Namer I.J. (2019). What Does Reduced FDG Uptake Mean in High-Grade Gliomas?. Clin. Nucl. Med..

[117] Martins R.G., Gonçalves L.G., Cunha N., Bugalho M.J. (2019). Metabolomic Urine Profile: Searching for New Biomarkers of SDHx-Associated Pheochromocytomas and Paragangliomas. J. Clin. Endocrinol. Metab..

[118] Ose J., Gigic B., Lin T., Liesenfeld D.B., Böhm J., Nattenmüller J., Scherer D., Zielske L., Schrotz-King P., Habermann N. (2019). Multiplatform Urinary Metabolomics Profiling to Discriminate Cachectic from Non-Cachectic Colorectal Cancer Patients: Pilot Results from the ColoCare Study. Metabolites.

[119] Taherkhani A., Nafar M., Arefi-Oskouie A., Broumandnia N., Parvin M., Mahmoudieh L., Kalantari S. (2019). Metabolomic Analysis of Membranous Glomerulonephritis: Identification of a Diagnostic Panel and Pathogenic Pathways. Arch. Med. Res..

[120] Akhbari P., Jaggard M.K., Boulangé C.L., Vaghela U., Graça G., Bhattacharya R., Lindon J.C., Williams H.R.T., Gupte C.M. (2019). Differences in the composition of hip and knee synovial fluid in osteoarthritis: A nuclear magnetic resonance (NMR) spectroscopy study of metabolic profiles. Osteoarthr. Cartil..

[121] Debik J., Euceda L.R., Lundgren S., Gythfeldt H.V.L., Garred Ø., Borgen E., Engebraaten O., Bathen T.F., Giskeødegård G.F. (2019). Assessing Treatment Response and Prognosis by Serum and Tissue Metabolomics in Breast Cancer Patients. J. Proteome Res..

[122] Bawadikji A.A., Teh C.H., Sheikh Abdul Kader M.A.B., Abdul Wahab M.J.B., Syed Sulaiman S.A., Ibrahim B. (2020). Plasma Metabolites as Predictors of Warfarin Outcome in Atrial Fibrillation. Am. J. Cardiovasc. Drugs.

[123] Righi V., Tarentini E., Mucci A., Reggiani C., Rossi M.C., Ferrari F., Casari A., Magnoni C. (2019). Field cancerization therapy with ingenol mebutate contributes to restoring skin-metabolism to normal-state in patients with actinic keratosis: A metabolomic analysis. Sci. Rep..

[124] Wildberg C., Masuch A., Budde K., Kastenmüller G., Artati A., Rathmann W., Adamski J., Kocher T., Völzke H., Nauck M. (2019). Plasma Metabolomics to Identify and Stratify Patients With Impaired Glucose Tolerance. J. Clin. Endocrinol. Metab..

[125] Huart J., Leenders J., Taminiau B., Descy J., Saint-Remy A., Daube G., Krzesinski J.M., Melin P., de Tullio P., Jouret F. (2019). Gut Microbiota and Fecal Levels of Short-Chain Fatty Acids Differ Upon 24-Hour Blood Pressure Levels in Men. Hypertension.

[126] Falegan O.S., Arnold Egloff S.A., Zijlstra A., Hyndman M.E., Vogel H.J. (2019). Urinary Metabolomics Validates Metabolic Differentiation Between Renal Cell Carcinoma Stages and Reveals a Unique Metabolic Profile for Oncocytomas. Metabolites.

[127] Liang J.H., Lin Y., Ouyang T., Tang W., Huang Y., Ye W., Zhao J.Y., Wang Z.N., Ma C.C. (2019). Nuclear magnetic resonance-based metabolomics and metabolic pathway networks from patient-matched esophageal carcinoma, adjacent noncancerous tissues and urine. World J. Gastroenterol..

[128] López-Garrido L., Bañuelos-Hernández A.E., Pérez-Hernández E., Tecualt-Gómez R., Quiroz-Williams J., Ariza-Castolo A., Becerra-Martínez E., Pérez-Hernández N. (2020). Metabolic profiling of serum in patients with cartilage tumours using ^1^H-NMR spectroscopy: A pilot study. Magn. Reson. Chem..

[129] Rodríguez-Tomàs E., Murcia M., Arenas M., Arguís M., Gil M., Amigó N., Correig X., Torres L., Sabater S., Baiges-Gayà G. (2019). Serum Paraoxonase-1-Related Variables and Lipoprotein Profile in Patients with Lung or Head and Neck Cancer: Effect of Radiotherapy. Antioxidants.

[130] Gawron K., Wojtowicz W., Łazarz-Bartyzel K., Łamasz A., Qasem B., Mydel P., Chomyszyn-Gajewska M., Potempa J., Mlynarz P. (2019). Metabolomic Status of The Oral Cavity in Chronic Periodontitis. In Vivo.

[131] Molinero N., Ruiz L., Milani C., Gutiérrez-Díaz I., Sánchez B., Mangifesta M., Segura J., Cambero I., Campelo A.B., García-Bernardo C.M. (2019). The human gallbladder microbiome is related to the physiological state and the biliary metabolic profile. Microbiome.

[132] Loras A., Martínez-Bisbal M.C., Quintás G., Gil S., Martínez-Máñez R., Ruiz-Cerdá J.L. (2019). Urinary Metabolic Signatures Detect Recurrences in Non-Muscle Invasive Bladder Cancer. Cancers.

[133] Lin H.T., Cheng M.L., Lo C.J., Lin G., Lin S.F., Yeh J.T., Ho H.Y., Lin J.R., Liu F.C. (2019). ^1^H Nuclear Magnetic Resonance (NMR)-Based Cerebrospinal Fluid and Plasma Metabolomic Analysis in Type 2 Diabetic Patients and Risk Prediction for Diabetic Microangiopathy. J. Clin. Med..

[134] Dalili N., Chashmniam S., Khoormizi S.M.H., Salehi L., Jamalian S.A., Nafar M., Kalantari S. (2020). Urine and serum NMR-based metabolomics in pre-procedural prediction of contrast-induced nephropathy. Intern. Emerg. Med..

[135] Lin C., Chen Z., Zhang L., Wei Z., Cheng K.K., Liu Y., Shen G., Fan H., Dong J. (2019). Deciphering the metabolic perturbation in hepatic alveolar echinococcosis: A ^1^H NMR-based metabolomics study. Parasit. Vectors.

[136] Mongan A.M., Lynam-Lennon N., Doyle S.L., Casey R., Carr E., Cannon A., Conroy M.J., Pidgeon G.P., Brennan L., Lysaght J. (2019). Visceral Adipose Tissue Modulates Radiosensitivity in Oesophageal Adenocarcinoma. Int. J. Med. Sci..

[137] Wijeyesekera A., Wagner J., De Goffau M., Thurston S., Rodrigues Sabino A., Zaher S., White D., Ridout J., Peters M.J., Ramnarayan P. (2019). Multi-Compartment Profiling of Bacterial and Host Metabolites Identifies Intestinal Dysbiosis and Its Functional Consequences in the Critically Ill Child. Crit. Care Med..

[138] Ghosh N., Choudhury P., Subramani E., Saha D., Sengupta S., Joshi M., Banerjee R., Roychowdhury S., Bhattacharyya P., Chaudhury K. (2019). Metabolomic signatures of asthma-COPD overlap (ACO) are different from asthma and COPD. Metabolomics.

[139] Fest J., Vijfhuizen L.S., Goeman J.J., Veth O., Joensuu A., Perola M., Männistö S., Ness-Jensen E., Hveem K., Haller T. (2019). Search for Early Pancreatic Cancer Blood Biomarkers in Five European Prospective Population Biobanks Using Metabolomics. Endocrinology.

[140] Klein C.F., Holle S.L.K., Andersen M.H., Pedersen A., Bundgaard H., Iversen K.K., Malmendal A. (2019). In-hospital metabolite changes in infective endocarditis-a longitudinal ^1^H NMR-based study. Eur. J. Clin. Microbiol. Infect. Dis..

[141] Del Coco L., Vergara D., De Matteis S., Mensà E., Sabbatinelli J., Prattichizzo F., Bonfigli A.R., Storci G., Bravaccini S., Pirini F. (2019). NMR-Based Metabolomic Approach Tracks Potential Serum Biomarkers of Disease Progression in Patients with Type 2 Diabetes Mellitus. J. Clin. Med..

[142] Loras A., Suárez-Cabrera C., Martínez-Bisbal M.C., Quintás G., Paramio J.M., Martínez-Máñez R., Gil S., Ruiz-Cerdá J.L. (2019). Integrative Metabolomic and Transcriptomic Analysis for the Study of Bladder Cancer. Cancers.

[143] Liu Z., Triba M.N., Amathieu R., Lin X., Bouchemal N., Hantz E., Le Moyec L., Savarin P. (2019). Nuclear magnetic resonance-based serum metabolomic analysis reveals different disease evolution profiles between septic shock survivors and non-survivors. Crit. Care.

[144] Chashmniam S., Kalantari S., Nafar M., Boroumandnia N. (2019). The metabolomics signature associated with responsiveness to steroid therapy in focal segmental glomerulosclerosis: A pilot study. Rev. Investig. Clin..

[145] Stryeck S., Gastrager M., Degoricija V., Trbušić M., Potočnjak I., Radulović B., Pregartner G., Berghold A., Madl T., Frank S. (2019). Serum Concentrations of Citrate, Tyrosine, 2- and 3- Hydroxybutyrate are Associated with Increased 3-Month Mortality in Acute Heart Failure Patients. Sci. Rep..

[146] Bund C., Guergova-Kuras M., Cicek A.E., Moussallieh F.M., Dali-Youcef N., Piotto M., Schneider P., Heller R., Entz-Werle N., Lhermitte B. (2019). An integrated genomic and metabolomic approach for defining survival time in adult oligodendrogliomas patients. Metabolomics.

[147] Rosado-Sánchez I., Rodríguez-Gallego E., Peraire J., Viladés C., Herrero P., Fanjul F., Gutiérrez F., Bernal E., Pelazas R., Leal M. (2019). Glutaminolysis and lipoproteins are key factors in late immune recovery in successfully treated HIV-infected patients. Clin. Sci..

[148] Alborghetti M.R., Correa M.E.P., Whangbo J., Shi X., Aricetti J.A., da Silva A.A., Miranda E.C.M., Sforca M.L., Caldana C., Gerszten R.E. (2019). Clinical Metabolomics Identifies Blood Serum Branched Chain Amino Acids as Potential Predictive Biomarkers for Chronic Graft vs. Host Disease. Front. Oncol..

[149] Onderwater G.L.J., Ligthart L., Bot M., Demirkan A., Fu J., van der Kallen C.J.H., Vijfhuizen L.S., Pool R., Liu J., Vanmolkot F.H.M. (2019). Large-scale plasma metabolome analysis reveals alterations in HDL metabolism in migraine. Neurology.

[150] Zhou Y.J., Li L.S., Sun J.L., Guan K., Wei J.F. (2019). ^1^H NMR-based metabolomic study of metabolic profiling for pollinosis. World Allergy Organ. J..

[151] Faitot F., Ruhland E., Oncioiu C., Besch C., Addeo P., Cicek A.E., Bachellier P., Namer I.J. (2019). Metabolomic profiling highlights the metabolic bases of acute-on-chronic and post-hepatectomy liver failure. HPB (Oxford).

[152] Yang Z., Liu Y., Ma L., Wen X., Ji H., Li K. (2019). Exploring potential biomarkers of early stage esophageal squamous cell carcinoma in pre- and post-operative serum metabolomic fingerprint spectrum using ^1^H-NMR method. Am. J. Transl. Res..

[153] Kim E.R., Kwon H.N., Nam H., Kim J.J., Park S., Kim Y.H. (2019). Urine-NMR metabolomics for screening of advanced colorectal adenoma and early stage colorectal cancer. Sci. Rep..

[154] Xu R., Zhu H., Zhang C., Shen G., Feng J. (2019). Metabolomic analysis reveals metabolic characteristics of children with short stature caused by growth hormone deficiency. Clin. Sci..

[155] Meoni G., Lorini S., Monti M., Madia F., Corti G., Luchinat C., Zignego A.L., Tenori L., Gragnani L. (2019). The metabolic fingerprints of HCV and HBV infections studied by Nuclear Magnetic Resonance Spectroscopy. Sci. Rep..

[156] Yanshole V.V., Yanshole L.V., Snytnikova O.A., Tsentalovich Y.P. (2019). Quantitative metabolomic analysis of changes in the lens and aqueous humor under development of age-related nuclear cataract. Metabolomics.

[157] Castiglione Morelli M.A., Iuliano A., Schettini S.C.A., Petruzzi D., Ferri A., Colucci P., Viggiani L., Cuviello F., Ostuni A. (2019). NMR metabolic profiling of follicular fluid for investigating the different causes of female infertility: A pilot study. Metabolomics.

[158] Amin A.M., Mostafa H., Arif N.H., Abdul Kader M.A.S., Kah Hay Y. (2019). Metabolomics profiling and pathway analysis of human plasma and urine reveal further insights into the multifactorial nature of coronary artery disease. Clin. Chim. Acta.

[159] Lorefice L., Murgia F., Fenu G., Frau J., Coghe G., Murru M.R., Tranquilli S., Visconti A., Marrosu M.G., Atzori L. (2019). Assessing the Metabolomic Profile of Multiple Sclerosis Patients Treated with Interferon Beta 1a by ^1^H-NMR Spectroscopy. Neurotherapeutics.

[160] Zacharias H.U., Altenbuchinger M., Schultheiss U.T., Samol C., Kotsis F., Poguntke I., Sekula P., Krumsiek J., Köttgen A., Spang R. (2019). A Novel Metabolic Signature To Predict the Requirement of Dialysis or Renal Transplantation in Patients with Chronic Kidney Disease. J. Proteome Res..

[161] Ahmed S., Dubey D., Chowdhury A., Chaurasia S., Guleria A., Kumar S., Singh R., Kumar D., Misra R. (2019). Nuclear magnetic resonance-based metabolomics reveals similar metabolomics profiles in undifferentiated peripheral spondyloarthritis and reactive arthritis. Int. J. Rheum. Dis..

[162] Khalid A., Siddiqui A.J., Ansari S.H., Musharraf S.G. (2019). Reflection of treatment proficiency of hydroxyurea treated β-thalassemia serum samples through nuclear magnetic resonance based metabonomics. Sci. Rep..

[163] Chen J.J., Xie J., Zeng L., Zhou C.J., Zheng P., Xie P. (2019). Urinary metabolite signature in bipolar disorder patients during depressive episode. Aging (Albany NY).

[164] Lin Y., Ma C., Bezabeh T., Wang Z., Liang J., Huang Y., Zhao J., Liu X., Ye W., Tang W. (2019). ^1^H NMR-based metabolomics reveal overlapping discriminatory metabolites and metabolic pathway disturbances between colorectal tumor tissues and fecal samples. Int. J. Cancer.

[165] Laíns I., Duarte D., Barros A.S., Martins A.S., Carneiro T.J., Gil J.Q., Miller J.B., Marques M., Mesquita T.S., Barreto P. (2019). Urine Nuclear Magnetic Resonance (NMR) Metabolomics in Age-Related Macular Degeneration. J. Proteome Res..

[166] Rawat A., Misra G., Saxena M., Tripathi S., Dubey D., Saxena S., Aggarwal A., Gupta V., Khan M.Y., Prakash A. (2019). ^1^H NMR based serum metabolic profiling reveals differentiating biomarkers in patients with diabetes and diabetes-related complication. Diabetes Metab. Syndr..

[167] Vignoli A., Tenori L., Giusti B., Takis P.G., Valente S., Carrabba N., Balzi D., Barchielli A., Marchionni N., Gensini G.F. (2019). NMR-based metabolomics identifies patients at high risk of death within two years after acute myocardial infarction in the AMI-Florence II cohort. BMC Med..

[168] Zhang P., Zhang W., Lang Y., Qu Y., Chen J., Cui L. (2019). ^1^H nuclear magnetic resonance-based metabolic profiling of cerebrospinal fluid to identify metabolic features and markers for tuberculosis meningitis. Infect. Genet. Evol..

[169] Vignoli A., Orlandini B., Tenori L., Biagini M.R., Milani S., Renzi D., Luchinat C., Calabrò A.S. (2019). Metabolic Signature of Primary Biliary Cholangitis and Its Comparison with Celiac Disease. J. Proteome Res..

[170] Harbaum L., Ghataorhe P., Wharton J., Jiménez B., Howard L.S.G., Gibbs J.S.R., Nicholson J.K., Rhodes C.J., Wilkins M.R. (2019). Reduced plasma levels of small HDL particles transporting fibrinolytic proteins in pulmonary arterial hypertension. Thorax.

[171] Romano F., Meoni G., Manavella V., Baima G., Mariani G.M., Cacciatore S., Tenori L., Aimetti M. (2019). Effect of non-surgical periodontal therapy on salivary metabolic fingerprint of generalized chronic periodontitis using nuclear magnetic resonance spectroscopy. Arch. Oral. Biol..

[172] Parra S., Lopez-Dupla M., Ibarretxe D., de Las Heras M., Amigó N., Català A., Benavent M., Garcés E., Navarro A., Castro A. (2019). Patients With Systemic Lupus Erythematosus Show an Increased Arterial Stiffness That is Predicted by IgM Anti-β(2) -Glycoprotein I and Small Dense High-Density Lipoprotein Particles. Arthritis Care Res. (Hoboken).

[173] Bahado-Singh R.O., Sonek J., McKenna D., Cool D., Aydas B., Turkoglu O., Bjorndahl T., Mandal R., Wishart D., Friedman P. (2019). Artificial intelligence and amniotic fluid multiomics: Prediction of perinatal outcome in asymptomatic women with short cervix. Ultrasound Obstet. Gynecol..

[174] Pocate-Cheriet K., Santulli P., Kateb F., Bourdon M., Maignien C., Batteux F., Chouzenoux S., Patrat C., Wolf J.P., Bertho G. (2020). The follicular fluid metabolome differs according to the endometriosis phenotype. Reprod. Biomed. Online.

[175] Jacyna J., Wawrzyniak R., Balayssac S., Gilard V., Malet-Martino M., Sawicka A., Kordalewska M., Nowicki Ł., Kurek E., Bulska E. (2019). Urinary metabolomic signature of muscle-invasive bladder cancer: A multiplatform approach. Talanta.

[176] Ijare O.B., Baskin D.S., Pichumani K. (2019). Ex Vivo ^1^H NMR study of pituitary adenomas to differentiate various immunohistochemical subtypes. Sci. Rep..

[177] Chen J., Ye C., Hu X., Huang C., Yang Z., Li P., Wu A., Xue X., Lin D., Yang H. (2019). Serum metabolomics model and its metabolic characteristics in patients with different syndromes of dyslipidemia based on nuclear magnetic resonance. J. Pharm. Biomed. Anal..

[178] Clendinen C.S., Gaul D.A., Monge M.E., Arnold R.S., Edison A.S., Petros J.A., Fernández F.M. (2019). Preoperative Metabolic Signatures of Prostate Cancer Recurrence Following Radical Prostatectomy. J. Proteome Res..

[179] Padayachee T., Khamiakova T., Louis E., Adriaensens P., Burzykowski T. (2019). The impact of the method of extracting metabolic signal from 1H-NMR data on the classification of samples: A case study of binning and BATMAN in lung cancer. PLoS ONE.

[180] Noorbakhsh H., Yavarmanesh M., Mortazavi S.A., Adibi P., Moazzami A.A. (2019). Metabolomics analysis revealed metabolic changes in patients with diarrhea-predominant irritable bowel syndrome and metabolic responses to a synbiotic yogurt intervention. Eur. J. Nutr..

[181] Nagana Gowda G.A., Raftery D. (2017). Whole Blood Metabolomics by ^1^H NMR Spectroscopy Provides a New Opportunity To Evaluate Coenzymes and Antioxidants. Anal. Chem..

[182] Gomez-Archila L.G., Palomino-Schatzlein M., Zapata-Builes W., Galeano E. (2021). Development of an optimized method for processing peripheral blood mononuclear cells for 1H-nuclear magnetic resonance-based metabolomic profiling. PLoS ONE.

[183] Hernandes V.V., Barbas C., Dudzik D. (2017). A review of blood sample handling and pre-processing for metabolomics studies. Electrophoresis.

[184] Beckonert O., Keun H.C., Ebbels T.M., Bundy J., Holmes E., Lindon J.C., Nicholson J.K. (2007). Metabolic profiling, metabolomic and metabonomic procedures for NMR spectroscopy of urine, plasma, serum and tissue extracts. Nat. Protoc..

[185] Bernini P., Bertini I., Luchinat C., Nincheri P., Staderini S., Turano P. (2011). Standard operating procedures for pre-analytical handling of blood and urine for metabolomic studies and biobanks. J. Biomol. NMR.

[186] Paglia G., Del Greco F.M., Sigurdsson B.B., Rainer J., Volani C., Hicks A.A., Pramstaller P.P., Smarason S.V. (2018). Influence of collection tubes during quantitative targeted metabolomics studies in human blood samples. Clin. Chim. Acta.

[187] Bi H., Guo Z., Jia X., Liu H., Ma L., Xue L. (2020). The key points in the pre-analytical procedures of blood and urine samples in metabolomics studies. Metabolomics.

[188] Emwas A.H., Roy R., McKay R.T., Ryan D., Brennan L., Tenori L., Luchinat C., Gao X., Zeri A.C., Gowda G.A. (2016). Recommendations and Standardization of Biomarker Quantification Using NMR-Based Metabolomics with Particular Focus on Urinary Analysis. J. Proteome Res..

[189] Lauridsen M., Hansen S.H., Jaroszewski J.W., Cornett C. (2007). Human urine as test material in 1H NMR-based metabonomics: Recommendations for sample preparation and storage. Anal. Chem..

[190] Soininen P., Kangas A.J., Wurtz P., Tukiainen T., Tynkkynen T., Laatikainen R., Jarvelin M.R., Kahonen M., Lehtimaki T., Viikari J. (2009). High-throughput serum NMR metabonomics for cost-effective holistic studies on systemic metabolism. Analyst.

[191] Lindon J.C., Nicholson J.K., Holmes E., Everett J.R. (2000). Metabonomics: Metabolic processes studied by NMR spectroscopy of biofluids. Concepts Magn. Reson..

[192] Soininen P., Kangas A.J., Wurtz P., Suna T., Ala-Korpela M. (2015). Quantitative serum nuclear magnetic resonance metabolomics in cardiovascular epidemiology and genetics. Circ. Cardiovasc. Genet..

[193] Ludwig C., Viant M.R. (2010). Two-dimensional J-resolved NMR spectroscopy: Review of a key methodology in the metabolomics toolbox. Phytochem. Anal..

[194] de Graaf R.A., Behar K.L. (2003). Quantitative 1H NMR spectroscopy of blood plasma metabolites. Anal. Chem..

[195] Mckay R.T. (2011). How the 1D-NOESY suppresses solvent signal in metabonomics NMR spectroscopy: An examination of the pulse sequence components and evolution. Concepts Magn. Reson. Part A.

[196] Vehtari A., Makinen V.P., Soininen P., Ingman P., Makela S.M., Savolainen M.J., Hannuksela M.L., Kaski K., Ala-Korpela M. (2007). A novel Bayesian approach to quantify clinical variables and to determine their spectroscopic counterparts in 1H NMR metabonomic data. BMC Bioinform..

[197] Crook A.A., Powers R. (2020). Quantitative NMR-Based Biomedical Metabolomics: Current Status and Applications. Molecules.

[198] Wishart D.S. (2008). Quantitative metabolomics using NMR. TrAC Trends Anal. Chem..

[199] Jacob D., Deborde C., Lefebvre M., Maucourt M., Moing A. (2017). NMRProcFlow: A graphical and interactive tool dedicated to 1D spectra processing for NMR-based metabolomics. Metabolomics.

[200] Norris M., Fetler B., Marchant J., Johnson B.A. (2016). NMRFx Processor: A cross-platform NMR data processing program. J. Biomol..

[201] Sousa S., Magalhaes A., Ferreira M. (2013). Optimized bucketing for NMR spectra: Three case studies. Chemom. Intell. Lab. Syst..

[202] Anderson P.E., Mahle D.A., Doom T.E., Reo N.V., DelRaso N.J., Raymer M.L. (2011). Dynamic adaptive binning: An improved quantification technique for NMR spectroscopic data. Metabolomics.

[203] Davis R.A., Charlton A.J., Godward J., Jones S.A., Harrison M., Wilson J.C. (2007). Adaptive binning: An improved binning method for metabolomics data using the undecimated wavelet transform. Chemom. Intell. Lab. Syst..

[204] De Meyer T., Sinnaeve D., Van Gasse B., Tsiporkova E., Rietzschel E.R., De Buyzere M.L., Gillebert T.C., Bekaert S., Martins J.C., Van Criekinge W. (2008). NMR-based characterization of metabolic alterations in hypertension using an adaptive, intelligent binning algorithm. Anal. Chem..

[205] Mercier P., Lewis M.J., Chang D., Baker D., Wishart D.S. (2011). Towards automatic metabolomic profiling of high-resolution one-dimensional proton NMR spectra. J. Biomol. NMR.

[206] Tulpan D., Leger S., Belliveau L., Culf A., Cuperlovic-Culf M. (2011). MetaboHunter: An automatic approach for identification of metabolites from 1(H)-NMR spectra of complex mixtures. BMC Bioinform..

[207] Xia J., Bjorndahl T.C., Tang P., Wishart D.S. (2008). MetaboMiner--semi-automated identification of metabolites from 2D NMR spectra of complex biofluids. BMC Bioinform..

[208] Ravanbakhsh S., Liu P., Bjorndahl T.C., Mandal R., Grant J.R., Wilson M., Eisner R., Sinelnikov I., Hu X., Luchinat C. (2015). Accurate, fully-automated NMR spectral profiling for metabolomics. PLoS ONE.

[209] Hao J., Liebeke M., Astle W., De Iorio M., Bundy J.G., Ebbels T.M. (2014). Bayesian deconvolution and quantification of metabolites in complex 1D NMR spectra using BATMAN. Nat. Protoc..

[210] van den Berg R.A., Hoefsloot H.C., Westerhuis J.A., Smilde A.K., van der Werf M.J. (2006). Centering, scaling, and transformations: Improving the biological information content of metabolomics data. BMC Genom..

[211] Emwas A.H., Saccenti E., Gao X., McKay R.T., Dos Santos V., Roy R., Wishart D.S. (2018). Recommended strategies for spectral processing and post-processing of 1D ^1^H-NMR data of biofluids with a particular focus on urine. Metabolomics.

[212] Dieterle F., Ross A., Schlotterbeck G., Senn H. (2006). Probabilistic Quotient Normalization as Robust Method to Account for Dilution of Complex Biological Mixtures. Application in 1H NMR Metabonomics. Anal. Chem..

[213] Craig A., Cloarec O., Holmes E., Nicholson J.K., Lindon J.C. (2006). Scaling and normalization effects in NMR spectroscopic metabonomic data sets. Anal. Chem..

[214] Jolliffe I.T., Cadima J. (2016). Principal component analysis: A review and recent developments. Philos. Trans. A Math. Phys. Eng. Sci..

[215] Gromski P.S., Muhamadali H., Ellis D.I., Xu Y., Correa E., Turner M.L., Goodacre R. (2015). A tutorial review: Metabolomics and partial least squares-discriminant analysis--a marriage of convenience or a shotgun wedding. Anal. Chim. Acta.

[216] Westerhuis J.A., van Velzen E.J., Hoefsloot H.C., Smilde A.K. (2010). Multivariate paired data analysis: Multilevel PLSDA versus OPLSDA. Metabolomics.

[217] Worley B., Powers R. (2013). Multivariate Analysis in Metabolomics. Curr. Metab..

[218] Zacharias H.U., Altenbuchinger M., Gronwald W. (2018). Statistical Analysis of NMR Metabolic Fingerprints: Established Methods and Recent Advances. Metabolites.

[219] Hazra A., Gogtay N. (2016). Biostatistics Series Module 2: Overview of Hypothesis Testing. Indian J. Dermatol..

[220] Hazra A., Gogtay N. (2016). Biostatistics Series Module 3: Comparing Groups: Numerical Variables. Indian J. Dermatol..

[221] Noble W.S. (2009). How does multiple testing correction work?. Nat. Biotechnol..

[222] Yu Z., Kastenmuller G., He Y., Belcredi P., Moller G., Prehn C., Mendes J., Wahl S., Roemisch-Margl W., Ceglarek U. (2011). Differences between human plasma and serum metabolite profiles. PLoS ONE.

[223] Denery J.R., Nunes A.A.K., Dickerson T.J. (2011). Characterization of Differences between Blood Sample Matrices in Untargeted Metabolomics. Anal. Chem..

[224] Sotelo-Orozco J., Chen S.Y., Hertz-Picciotto I., Slupsky C.M. (2021). A Comparison of Serum and Plasma Blood Collection Tubes for the Integration of Epidemiological and Metabolomics Data. Front. Mol. Biosci..

[225] Lesche D., Geyer R., Lienhard D., Nakas C.T., Diserens G., Vermathen P., Leichtle A.B. (2016). Does centrifugation matter? Centrifugal force and spinning time alter the plasma metabolome. Metabolomics.

[226] Jobard E., Tredan O., Postoly D., Andre F., Martin A.L., Elena-Herrmann B., Boyault S. (2016). A Systematic Evaluation of Blood Serum and Plasma Pre-Analytics for Metabolomics Cohort Studies. Int. J. Mol. Sci..

[227] Ammerlaan W., Trezzi J.P., Mathay C., Hiller K., Betsou F. (2014). Method validation for preparing urine samples for downstream proteomic and metabolomic applications. Biopreserv. Biobank..

[228] Snytnikova O.A., Khlichkina A.A., Sagdeev R.Z., Tsentalovich Y.P. (2019). Evaluation of sample preparation protocols for quantitative NMR-based metabolomics. Metabolomics.

[229] McHugh C.E., Flott T.L., Schooff C.R., Smiley Z., Puskarich M.A., Myers D.D., Younger J.G., Jones A.E., Stringer K.A. (2018). Rapid, Reproducible, Quantifiable NMR Metabolomics: Methanol and Methanol: Chloroform Precipitation for Removal of Macromolecules in Serum and Whole Blood. Metabolites.

[230] Sheedy J.R., Ebeling P.R., Gooley P.R., McConville M.J. (2010). A sample preparation protocol for 1H nuclear magnetic resonance studies of water-soluble metabolites in blood and urine. Anal. Biochem..

[231] Lane A. (2012). Principles of NMR for Applications in Metabolomics.

[232] Mishra P., Pandey C.M., Singh U., Gupta A., Sahu C., Keshri A. (2019). Descriptive statistics and normality tests for statistical data. Ann. Card. Anaesth..

[233] Vinaixa M., Samino S., Saez I., Duran J., Guinovart J.J., Yanes O. (2012). A Guideline to Univariate Statistical Analysis for LC/MS-Based Untargeted Metabolomics-Derived Data. Metabolites.

[234] Breiman L. (2001). Statistical Modeling: The Two Cultures (with comments and a rejoinder by the author). Stat. Sci..

[235] Malsagova K., Kopylov A., Stepanov A., Butkova T., Izotov A., Kaysheva A. (2020). Dried Blood Spot in Laboratory: Directions and Prospects. Diagnostics.

[236] Lei B.U.W., Prow T.W. (2019). A review of microsampling techniques and their social impact. Biomed. Microdevices.

[237] Wishart D.S. (2019). NMR metabolomics: A look ahead. J. Magn. Reson..

[238] Emwas A.H., Roy R., McKay R.T., Tenori L., Saccenti E., Gowda G.A.N., Raftery D., Alahmari F., Jaremko L., Jaremko M. (2019). NMR Spectroscopy for Metabolomics Research. Metabolites.

[239] Wishart D.S., Cheng L.L., Copie V., Edison A.S., Eghbalnia H.R., Hoch J.C., Gouveia G.J., Pathmasiri W., Powers R., Schock T.B. (2022). NMR and Metabolomics-A Roadmap for the Future. Metabolites.

[240] Dey A., Charrier B., Martineau E., Deborde C., Gandriau E., Moing A., Jacob D., Eshchenko D., Schnell M., Melzi R. (2020). Hyperpolarized NMR Metabolomics at Natural 13C Abundance. Anal. Chem..

[241] Hackl M., Tauber P., Schweda F., Zacharias H.U., Altenbuchinger M., Oefner P.J., Gronwald W. (2021). An R-Package for the Deconvolution and Integration of 1D NMR Data: MetaboDecon1D. Metabolites.

[242] Migdadi L., Lambert J., Telfah A., Hergenroder R., Wohler C. (2021). Automated metabolic assignment: Semi-supervised learning in metabolic analysis employing two dimensional Nuclear Magnetic Resonance (NMR). Comput. Struct. Biotechnol J..

[243] Markley J.L., Dashti H., Wedell J.R., Westler W.M., Eghbalnia H.R. (2019). Tools for Enhanced NMR-Based Metabolomics Analysis. Methods Mol. Biol..

