# Supplementary material for: Systematic Review of NMR-Based Metabolomics Practices in Human Disease Research
Source: Metabolites. 2022 Oct 12;12(10):963. doi: 10.3390/metabo12100963 (PMC9609461; doi:10.3390/metabo12100963)
Supplement: Supplementary file 1 [file metabolites-12-00963-s001.zip › Supplementary Table.pdf]

Supplementary Table 1: Summary table for serum, plasma, and urine.

	Plasma		Serum		Urine			Plasma		Serum		Urine	
	n	%	n	%	n	%		n	%	n	%	n	%
**Sample collection**													
**Sample collection collected under fasting**							**Urine collection condition**						
Fasted	12	35%	35	52%	17	37%	First Morning Void					19	41%
Not Reported	19	56%	28	42%	29	63%	Not Reported					21	46%
Non-Fasted	2	6%	3	4%			24-Hour		NA			2	4%
Some Fasted	1	3%	1	1%			Random					3	7%
**Blood collection tube**							Spot					1	2%
Not Reported	14	41%	46	69%	30	65%	**Midstream urine collected**						
Heparin	5	15%					No	NA		NA		36	78%
EDTA	14	41%		NA			Yes					10	22%
Sodium Citrate	1	3%					**Sample centrifuged at either collection or preparation step**						
No Additive			13	19%			Yes	23	68%	46	69%	29	63%
Gel Separator	NA		5	7%	NA		Not Reported/None	11	32%	21	31%	17	37%
Silica			3	4%			**Reported centrifugation parameters at collection**						
Sterile cups/containers					14	30%	Yes	13	38%	34	51%	15	33%
Coated With Ascorbic Acid		NA			1	2%	Not Reported/None	21	62%	33	49%	31	67%
Coated With NaN_3					1	2%	**Sample storage temperature**						
**Blood standing time (min)**							-80	22	65%	47	70%	33	72%
Not Reported	32	94%	45	67%			Not Reported	11	32%	14	21%	9	20%
30	2	6%	8	12%			-20	1	3%	1	1%	2	4%
60			4	6%			-70			3	4%	1	2%
30-40			3	4%			-60			1	1%	1	2%
<120			1	1%			-81			1	1%		
<60			1	1%	NA		**Sample collection reference**						
15			1	1%			Not Reported	30	88%	58	87%	42	91%
240			1	1%			Dona (2014)	1	3%	1	1%	1	2%
30-120			1	1%			Beckonert (2007)	1	3%	2	3%	2	4%
30-60			1	1%			Soininen (2015)	1	3%	2	3%		
30-90			1	1%			Bernini (2011)			3	4%		
							Other	1	3%	1	1%	1	2%
**Sample Preparation**													
**Sample preparation step**							**Chemical shift reference used in NMR buffer**						
No Preparation	27	79%	56	84%	44	96%	Not Reported/None	14	41%	26	39%	6	13%
Ultrafiltration	5	15%	5	7%	2	4%	TSP	15	44%	29	43%	36	78%
Metabolite Extraction	2	6%	6	9%			DSS	4	12%	8	12%	3	7%
**NMR buffer type**							Formate	1	3%	2	3%		
Phosphate	8	24%	15	22%	13	28%	Fumarate			1	1%		
Sodium Phosphate	15	44%	30	45%	13	28%	Maleic Acid			1	1%	1	2%
Potassium Phosphate	4	12%	1	1%	18	39%	**Sample preparation reference**						
Not Reported	5	15%	11	16%			Not Reported	18	53%	37	55%	25	54%
Saline	1	3%	2	3%	1	2%	Dona (2014)	3	9%	4	6%	4	9%
D_2O	1	3%	8	12%			Soininen (2015)	5	15%	8	11%		
Chenomx ISTD					1	2%	Beckonert (2007)			6	9%	4	9%
**pH of NMR buffer**							Bernini (2011)			6	9%	2	4%
7.4	14	41%	34	51%	26	57%	Other	8	21%	6	6%	11	22%
Not Reported	17	50%	30	45%	10	22%	**NaN_3 included in NMR buffer**						

7	2	6%	3	4%	7	15%	Not Reported/None	24	71%	51	76%	27	59%
6.5					1	2%	Yes	10	29%	16	24%	19	41%
7.2					1	2%	**Reported centrifugation parameters prior to sample preparation**						
7.47	1	3%			1	2%	Yes	14	41%	16	24%	20	43%
							Not Reported/None	20	59%	51	76%	26	57%
**NMR spectra acquisition**													
**NMR frequency**							**Nightingale Health**						
600	18	53%	36	54%	23	50%	Yes	5	15%	9	13%	NA	
700	2	6%	1	1%	2	4%	**NMR Processing Software**						
500	6	18%	9	13%	15	33%	Mnova (Mestrelab)	2	6%	10	15%	9	20%
Not Reported	7	21%	12	18%			Topspin (Bruker)	13	38%	23	34%	18	39%
400, 900	1	3%					Chenomx	2	6%	6	9%	5	11%
800			8	12%	3	7%	MAGMET	1	3%				
750			1	1%			KNIME (KIMBLE)	1	3%				
400					3	7%	Not Reported	12	35%	19	28%	7	15%
**Pulse sequence**							MestReC (Mestrelab)	1	3%			1	2%
NOESY	17	50%	17	25%	34	74%	ACD/Labs	1	3%				
CPMG	18	53%	43	64%	10	22%	Topspin and R	1	3%				
Diffusion Edited	4	12%	8	12%			NMRProcFlow			1	1%		
2D J-Resolved	6	18%	4	6%	3	7%	MATLAB			5	7%	6	12%
**Other pulse sequences**							PERCH NMR Software (Bruker)			1	1%		
zg30	2	6%	1	1%	1	2%	iNMR			1	1%		
p3919g			2	3%	1	2%	NMRPipe			1	1%		
zgesgp			2	3%	2	4%	**Acquisition Temperature**						
zgcppr			1	1%			300	9	26%	4	6%	18	39%
zgpr					1	2%	311	1	3%				
**Acquisition Reference**							298	2	6%	20	30%	12	26%
Not Reported	18	53%	45	67%	30	65%	310	7	21%	16	24%		
Dona (2014)	2	6%	2	3%	3	7%	Not Reported	13	38%	25	37%	12	26%
Soininen (2015)	5	15%	7	10%			299	1	3%				
Mallol (2015)	1	3%	2	3%			297	1	3%	1	1%	1	2%
Beckonert (2007)	1	3%	6	9%	3	7%	295			1	1%		
Bernini (2011)			1	1%			293					1	2%
Other	5	15%	4	4%	10	20%	301					1	2%
							303					1	2%
**Data type**													
**Data Type**							**Profiling Software**						
Bins and Concentrations	11	32%	14	21%	12	26%	Bruker IVDr (Bruker)	4	12%	1	1%	1	2%
Metabolite Concentrations only	14	41%	29	43%	9	20%	Chenomx	10	29%	15	22%	13	28%
Spectral Bins only	9	26%	24	36%	25	54%	MAGMET	1	3%				
**Binning Software**							Nightingale Health	5	15%	9	13%		
Not Reported	16	47%	34	51%	11	24%	Lipid/Lipoprotein Profiling	1	3%	3	4%		
AMIX	9	26%	16	24%	10	22%	Not Reported	10	29%	28	42%	26	57%
Mnova (Mestrelab)	3	9%	8	12%	7	15%	MATLAB	1	3%				
MATLAB	1	3%	4	5%	9	20%	BATMAN	1	3%				
ACD/Labs	2	6%					Lipoprofile Test	1	3%				
Chenomx	1	3%			1	2%	Axinon LipoFIT-S100			1	1%		
Intelligent Binning	1	3%					Mnova (MestreLab)			1	1%	2	4%

BATMAN	1	3%				2%	AMIX (Bruker)			5	7%		
AssureNMR (Bruker)			1	1%	1		PERCH Solutions (Bruker)			2	3%		
NMRProcFlow			1	1%		4%	STOCSY			1	1%	1	2%
Recursive Segment Wise Peak Alignment			1	1%	2	2%	MetaboMiner			1	1%		
Dynamic Adaptive Binning			1	1%	1		VnmrJ Software (Agilent)					1	2%
Optimised Bucketing			1	1%		4%	MetaboHunter					1	2%
Perl Script					2	2%	Bayesian Information Criterion					1	2%
dataChord Spectrum Miner					1	2%							
KnowItAll Software					1								
**Data and pathway analysis**													
**Normalisation**							**Univariate analyses (UVA)**						
Total Sum	10	29%	24	36%	13	28%	Student's t	9	26%	29	43%	12	26%
Not Reported/None	19	56%	29	43%	13	28%	Mann Whitney U	27	79%	44	66%	22	48%
Chemical shift reference	2	6%	5	7%	1	2%	Kruskal-Wallis	4	12%	3	4%	4	9%
Median	2	6%			2	4%	Wilcoxon signed rank	2	6%	7	10%	3	7%
Formate	1	3%					Welsh's t	1	3%				
PQN			7	10%	11	24%	ANOVA	5	15%	14	21%	4	9%
Mean			1	1%			ANCOVA	1	3%	1	1%		
Multiply by 3			1	1%			Log Rank Test			1	1%		
Creatinine					4	9%	AUROC	9	26%	18	27%	13	28%
Quantile					2	4%	**Normality Test**						
**Scaling**							Not Reported/None	31	91%	60	90%	42	91%
Pareto	8	24%	12	18%	4	9%	Shapiro-Wilk	1	3%	1	1%	3	7%
Unit variance	9	26%	17	25%	19	41%	Kolmogorov-Smirnoff	2	6%	4	6%	1	2%
Not Reported/None	16	47%	36	54%	21	46%	D'Agostino-Pearson			1	1%		
Mean	1	3%	2	3%	2	4%	Histogram			1	1%		
**Transformation**							**Multiple Testing Correction**						
Not reported/None	24	71%	54	81%	37	80%	Not reported/None	23	68%	39	58%	25	54%
Log_{10}	8	24%	12	18%	8	17%	FDR	1	3%	5	7%	1	2%
Log_e	1	3%					Holm	2	6%				
Log_2	1	3%	1	1%			Benjamini-Yekutieli	1	3%				
Cubic Root					1	2%	Bonferroni	3	9%	5	7%	7	15%
**Analysis Type**							Benjamini-Hochberg	3	9%	12	17%	10	22%
MVA and UVA	24	71%	48	72%	35	76%	Westfall-Young	1	3%	1	1%		
MVA	9	26%	6	9%	6	13%	Dunn			1	1%	2	4%
UVA	1	3%	13	19%	5	11%	Tukey			4	6%	1	2%
**Multivariate analyses (MVA)**							**Correlation**						
Hotelling's T^2	4	12%	4	6%	5	11%	Not reported/None	25	74%	48	72%	35	76%
PCA	16	47%	27	40%	17	37%	Spearman	3	9%	7	10%	5	11%
PLS-DA	11	32%	26	39%	17	37%	Pearson	6	18%	10	15%	6	13%
OPLS-DA	19	56%	42	63%	24	52%	**Statistical Software**						
Logistics Regression	21	62%	62	93%	40	87%	SPSS	13	38%	18	27%	13	28%
Random Forest	6	18%	5	7%	4	9%	SIMCA	12	35%	14	21%	21	46%
HCA	2	6%	1	4%	2	4%	R	9	26%	14	21%	9	20%
t-SNE	1	3%					GraphPad	4	12%	13	19%	2	4%
K-means	2	6%			1	2%	MATLAB	3	9%	7	10%	4	9%
Linear Regression	1	3%			1	2%	MetaboAnalyst	4	12%	17	25%	9	20%
Cox Regression	3	9%	3	4%			**Pathway Analysis**						

Global Test Analysis	1	3%					Not Reported/None	25	74%	47	70%	25	54%
Factor Analysis	1	3%			1	2%	Metaboanalyst	5	15%	16	24%	15	32%
SVM	2	6%	2	2%	1	1%	KEGG	3	9%	3	4%	2	4%
sPLS-DA			1	1%			Cytoscape	1	3%				
Disease State Index Classifier			1	1%			STITCH	1	3%				
Multilevel PLS			4	5%	1	2%	MBRole			1	1%	1	2%
							MetScape (Cytoscape)					1	2%
							IMPaLA					3	6%

Please note some percentages do not add up to 100% as some studies used multiple methods and software.
